# Lipid droplets as stress-buffering organelles in cancer cell homeostasis

**DOI:** 10.1038/s42003-026-10566-5

**Published:** 2026-06-29

**Authors:** Ruolun Wei, Claudia K. Petritsch

**Affiliations:** 1 Stanford University School of Medicine, Stanford, CA USA; 2 Department of Neurosurgery, Stanford University School of Medicine, Stanford, CA USA; 3 Department of Neurology and Neurosciences, Stanford University School of Medicine, Stanford, CA USA; 4 Stanford Cancer Institute, Stanford University School of Medicine, Stanford, CA USA; 5 Cancer Model Development Center, Stanford University School of Medicine, Stanford, CA USA

**Keywords:** Cancer metabolism, Tumour heterogeneity

## Abstract

Lipid droplets (LDs) are dynamic organelles that support homeostasis and shield cancer cells from stress to promote cancer cell survival, progression, and therapy escape. Here, we summarize recent findings demonstrating LD crosstalk with other organelles and the immune/stromal compartments aimed at buffering metabolic, oxidative, proteotoxic, and lipotoxic stress and stress from cancer treatment and the anti-tumor immune response. We briefly summarize current experimental approaches, including imaging, biochemical, and functional approaches, used to study LD biology. Together, these findings place LDs at the center of cancer cell homeostasis and highlight their emerging potential as translational targets in cancer therapy.

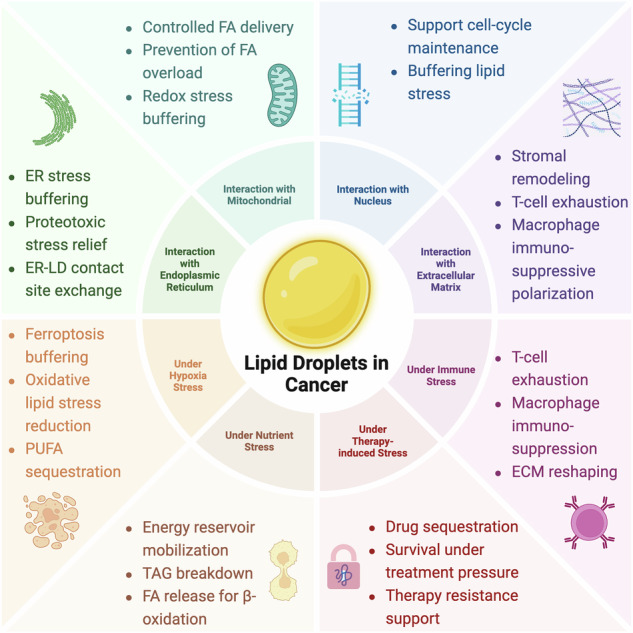

## Abstract (web summary)

A review summarizes recent findings demonstrating the extensive remodeling of lipid droplets (LDs) in cancer and their roles in buffering cellular stress, including from cancer therapy

## Introduction

Lipid droplets (LDs) were first described by Antonie van Leeuwenhoek in 1674, during his microscopic observations of oil droplets in milk, making them one of the earliest identified cellular compartments. What distinguishes LDs from other membrane-bound organelles is their unique structure: they are surrounded by a phospholipid monolayer, as opposed to the lipid bilayers that enclose other organelles. The core of an LD typically consists of neutral lipids such as triacylglycerols (TAGs), cholesterol, and sterols^1–3^. Originally, LDs evolved in eukaryotes as specialized energy storage compartments that sequester excess lipids during periods of abundant nutrients and release them when resources are scarce. While LDs are commonly found in eukaryotic cells, they are also evolutionarily ancient and conserved across a wide range of organisms. For a long time, LDs were regarded as inert lipid storage compartments, but with the discovery of proteins anchored to their lipid monolayer, they became recognized as functional organelles with a role in managing lipid metabolism, and connecting different signaling pathways^4^. Moreover, LDs safeguard against various types of cellular stress and maintain energy homeostasis^4^. Given that cancer cells face a vast variety of stressors and often contain numerous LDs, it will be critical to illuminate the specific mechanisms through which LDs interact with various organelles. LDs facilitate cell proliferation, migration, and survival through various mechanisms that are highly specific to cancer cell type^5,6^. In this review, we explore the role of LDs in buffering stress from multiple sources, including nutrient deprivation, hypoxia, endoplasmic reticulum stress, mitochondrial oxidative stress, nuclear stress, and therapy-induced insults. By doing so, LDs play a crucial part in promoting cancer cell survival and progression, elevating them from mere lipid storage depots to pivotal players in cancer cell adaptation^7,8^ (Graphic Abstract). The specific methods employed to study LD functions significantly influence both data generation and interpretation. Therefore, we will also provide a brief overview of experimental approaches and discuss how to utilize them for a more critical interpretation of the data in this field.

## Review

### LDs biosynthesis in cancer

**Fig. 1:**
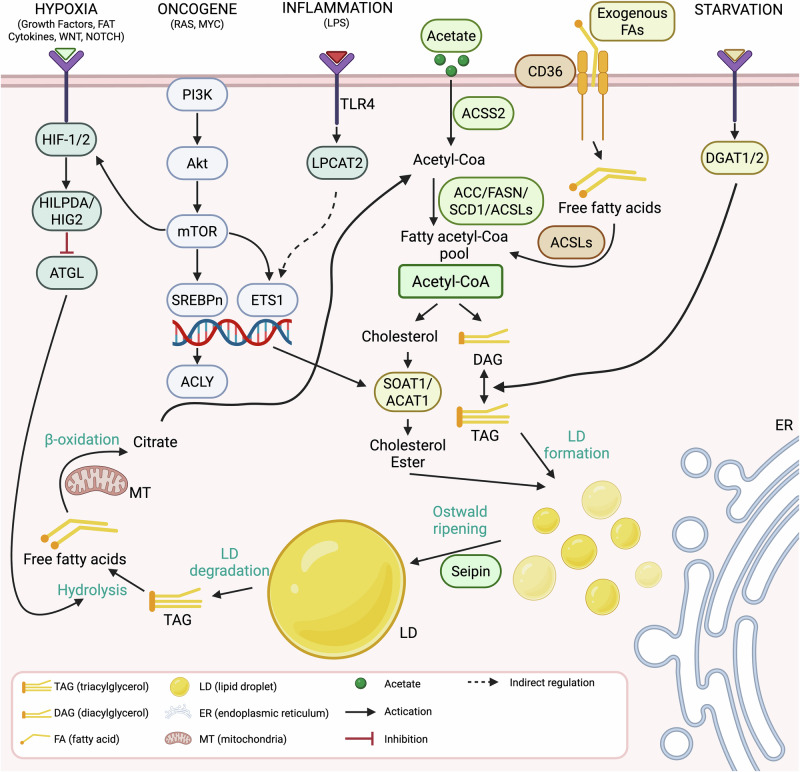
Cancer-associated lipid droplet formation and accumulation. Schematic of oncogenic and stress-related pathways that promote lipid droplet LD formation in cancer. Phosphoinositide 3-kinase (PI3K)–protein kinase B (Akt)–mechanistic target of rapamycin (mTOR), hypoxia-inducible factor 1/2 (HIF-1/2)–hypoxia-inducible lipid droplet-associated protein/hypoxia-inducible gene 2 (HILPDA/HIG2)–adipose triglyceride lipase (ATGL), Toll-like receptor 4 (TLR4)–lysophosphatidylcholine acyltransferase 2 (LPCAT2), cluster of differentiation 36 (CD36)-mediated lipid uptake, acetyl-CoA synthetase 2 (ACSS2)-mediated acetate utilization, and diacylglycerol O-acyltransferase 1/2 (DGAT1/2)- or sterol O-acyltransferase 1/acetyl-CoA acetyltransferase 1 (SOAT1/ACAT1)-dependent neutral lipid synthesis converge to expand triacylglycerol (TAG) and cholesterol ester pools and support LD biogenesis, growth, and turnover. Additional abbreviations: fatty acid (FA), fatty acyl-CoA pool, acetyl-CoA, acetyl-CoA carboxylase (ACC), fatty acid synthase (FASN), stearoyl-CoA desaturase 1 (SCD1), acyl-CoA synthetase long-chain family members (ACSLs), diacylglycerol (DAG), endoplasmic reticulum (ER), mitochondria (MT), lipopolysaccharide (LPS), rat sarcoma viral oncogene homolog (RAS), and MYC proto-oncogene (MYC). Created with BioRender.com.

LDs arise de novo from the endoplasmic reticulum (ER) in response to excess lipids or various stimuli and stresses^8,9^. Neutral lipids, including TAGs and cholesteryl esters (CEs), accumulate within the ER bilayer and bud toward the cytosol to form nascent LDs encased by a phospholipid monolayer^10,11^. Neutral lipid synthesis proceeds through two main branches that converge on LD formation. In the fatty acid (FA) synthesis pathway, ATP-citrate lyase (ATP-citrate lyase) generates acetyl-CoA from citrate, acetyl-CoA carboxylase (ACC) converts acetyl-CoA into malonyl-CoA, and fatty acid synthase (FASN) produces saturated FAs such as palmitate^12^. These FAs are then activated to acyl-CoA and incorporated into glycerolipids. In the TAG synthesis pathway, intermediates such as phosphatidic acid and diacylglycerol (DAG) are generated through enzymes including AGPAT2 and Lipin-1. Diacylglycerol O-acyltransferases (DGAT1/2) catalyze the final step from DAG to TAG^13,14^. In parallel, sterol O-acyltransferase 1 (SOAT1) esterifies free cholesterol to generate CEs^15^. Together, TAGs and CEs accumulate within the ER bilayer and provide the neutral lipid core that seeds nascent LD formation. Although both TAGs and CEs contribute to the neutral lipid core, they play distinct roles in cancer metabolism: TAGs mainly support FA storage, buffering, and, at later stages, also mobilization for energy use. CEs, on the other hand, are more closely linked to cholesterol storage and cholesterol-dependent membrane formation and signaling^15,16^. Thus, multiple proteins collaborate to form LDs: Diacylglycerol O-acyltransferases 1 and 2 and SOAT1, encoded by *DGAT1/2* and *SOAT1*, respectively, generate neutral lipids^17^, SEIPIN encoded by *BSCL2,* nucleates and stabilizes ER–LD junctions^18^, while fat storage-inducing transmembrane protein 2 (FIT2), encoded by *FITM2*, coordinates ER tubules and cytoskeletal elements to promote LD emergence and growth^19^. Lastly, dedicated LD surface proteins called perilipins subsequently regulate access by lipases and biosynthetic enzymes^20^. Beyond simple budding, LD size expansion may also be explained by biophysical models such as coalescence and Ostwald ripening, which have been proposed to contribute to LD enlargement in certain cellular settings (Fig. 1)^21,22^.

Although LDs are highly heterogeneous in cancer, ranging from small to enlarged droplets and differing in composition and protein cargo, increased LD enrichment remains a recurrent feature across many cancer types and aggressive cancer states^9^. Cancer cells promote LD formation through lipid metabolic reprogramming driven by oncogenic signaling and microenvironmental stress through multiple mechanisms: (i) Oncogenic PI3K/AKT/mTOR signaling activates SREBP, which in turn upregulates de novo lipogenesis through expression and functional activation of downstream target genes (*ATP-citrate lyase/ACC/FASN*), and cholesteryl esterification, expanding the neutral lipid pool that feeds LD biogenesis, particularly in Akt-driven tumor contexts such as glioblastoma^23–25^; Vice versa, inhibition of this axis curtails lipogenesis and increases vulnerability to lipid peroxidation-induced cell death, primarily known as ferroptosis, as shown in pancreatic cancer^26^. (ii) Hypoxia (HIF-1/2) promotes lipid storage by upregulating HILPDA/HIG2, a lipid-droplet protein that inhibits the adipose triglyceride lipase ATGL, thereby suppressing lipolysis and promoting TAG accumulation in hypoxic cancer settings models, including clear cell renal cell carcinoma^27,28^. (iii) Rapid proliferation across various types of cancer cells induces nutrient stress and autophagy, which release FAs and channel them into LDs via DGAT1/2. This predominantly expands the TAG pool, thereby buffering lipotoxicity and staging FAs for later oxidation, a phenomenon observed in diverse types of cancer including melanoma^5,29^. (iv) Cancer cells expand their neutral lipid pool via accumulation of CEs via the ETS1–SOAT1 regulatory axis, which correlates with enhanced invasion, metastatic potential, and poor prognosis, as shown in oral squamous cell carcinoma^30^. Consistent with this, LD formation has also been linked to metastatic behavior in specific cancer contexts; for example, acidosis-driven LD accumulation can support epithelial-mesenchymal transition and promote metastatic spreading through transforming growth factor beta2-dependent FA uptake and activation of storage pathways in various cancer models^31^. Besides endogenous synthesis, many cancers import lipids to build LDs. CD36, upregulated in breast cancer, gastric cancer, and liver metastasis, enhances FA uptake and supports LD accumulation, promoting cancer aggressiveness^32–35^. In parallel, cancer cells exploit acetate as a carbon source: Acetyl-CoA synthetase 2 converts acetate to acetyl-CoA for lipogenesis, particularly under hypoxia or lipid limitation, supporting LD biogenesis and growth^36–38^. These uptake and salvage routes complement SREBP-driven de novo lipogenesis and ensure an alternative neutral lipid supply for LD formation (Fig. 1).

### Structural and functional remodeling of LDs in cancer

LDs universally contain a neutral lipid core primarily composed of TAGs and CEs, with occasional retinyl ester and ceramide storage depending on the cell type^16,39–41^. This hydrophobic core is enveloped by a phospholipid monolayer, rich in phosphatidylcholine, phosphatidylethanolamine (PE), and other phospholipids^42–44^. In certain specialized cell types, such as retinoid-storing cells, this core can also contain vitamin-derived retinyl esters and other hydrophobic compounds^45^. Related retinoid-associated signals have also been detected in LDs of breast cancer cells under retinoid-treated conditions^46^. Decorated with a cohort of structural proteins, most notably the Perilipin, Adipophilin, and TIP47 (PAT) family proteins (e.g., PLIN1–5), this protein coat regulates LD stability and lipase access^47–50^. Beyond these stable building blocks of lipids and coat proteins, a dynamic group of enzymes (e.g., acyltransferases, lipases) and interactome constituents facilitate lipid flux and regulate LD-mediated signaling^51–54^.

In cancer cells, LDs undergo significant compositional remodeling to support the needs of malignant behaviors^55,56^. Elevated levels of TAGs and CEs in the core fuel rapid proliferation of cancer cells while protecting them against oxidative stress^15,51,57,58^. In cancer cells, the protein complement of LDs is remodeled in a context-dependent manner. In particular, DGAT1 and DGAT2 are frequently upregulated or functionally engaged in cancer cells with high lipid flux, where they support TAG synthesis, LD expansion, and stress adaptation. DGAT1-dependent LD biogenesis has been linked to cancer cell growth and oxidative stress buffering in melanoma and glioblastoma models, whereas DGAT2-driven LD biogenesis has been associated with increased migration, radiosensitivity, and aggressive phenotypes in breast cancer and other cancer models^59,60^. Moreover, LDs can recruit enzymes involved in the lipid production of signaling molecules in specific cancer contexts. Cyclooxygenase-2 (COX-2) can convert arachidonic acid into the prostaglandin endoperoxides prostaglandin G2 and H2, which are subsequently undergoing further processing by downstream prostaglandin synthases into prostaglandins such as prostaglandin E2^61^. In colon cancer cells, lipid bodies act as reservoirs of COX-2 and as sites of prostaglandin E2 synthesis, supporting the idea that at least part of the eicosanoid production can occur directly at LD-associated structures rather than being confined only to nearby ER membranes^62^. Consistent with this, coordinated induction of LD biogenesis and the COX-2 pathway has also been reported in patient samples obtained from Barrett’s esophagus and esophageal adenocarcinoma, both risk-associated with obesity. In contrast, no increase was observed in esophageal squamous cell carcinoma, which is risk-associated with tobacco and alcohol consumption but not obesity. Moreover, experimentally increasing LD amounts in a non-tumor esophageal cell line upregulates expression of COX-2, CXCL-8, and IL-8 secretion, creating a pro-inflammatory microenvironment^62^. More recent work furthermore suggests that LD biogenesis can facilitate production of growth-promoting lipid signals in human cancer cell models, including of breast cancer, cervical cancer, and lung cancer^63,64^. COX2 inhibition is being evaluated clinically in metastatic colorectal cancer and may prevent progression, but only in a specific patient subgroup that has yet to be determined^65^. Together, these observations indicate that LD-associated eicosanoid production is functionally relevant and may orchestrate pro-tumor inflammatory programs in a context-dependent manner rather than universally across all cancers. In addition, the observation that LD composition can vary across cancer regions and cellular states indicates that LD remodeling is spatially heterogeneous^56^. More research is needed to determine whether these changes reflect both remodeling of LD-associated protein cargo and broader lipid metabolic rewiring in specific cancer cell types. Such studies are needed to further explore lipid uptake, enhanced de novo lipogenesis, and reduced LD catabolism as both potential drivers for cancer progression and as therapeutic vulnerabilities.

### The role of LDs in buffering nutrient stress in cancer

Cancer cells constantly face stress from nutrient deprivation, and LD are critical organelles in adapting to this stress by shifting from building and storing lipids to burning them for providing energy^64,66–68^. This mobilization of LDs under energy deficit is tightly regulated by nutrient-sensing pathways, in particular the AMP-activated kinase (AMPK), which is activated by low ATP levels^69^. Activated AMPK promotes autophagy and lipophagy, enhancing the delivery of LDs to lysosomes to release stored FAs into the cytoplasm^70^. Activated AMPK also inhibits FA synthesis and promotes FA oxidation by phosphorylating and inhibiting acetyl-CoA carboxylase (ACC), lowering malonyl-CoA to activate CPT1A, a key regulator of FA breakdown into energy sources, including ATP and NADH^71,72^. This catabolic response is, however, counterbalanced by oncogenic PI3K–AKT–mTOR signaling, a universal driver of cancer cell growth, which favors anabolic lipid metabolism and suppresses autophagy under nutrient-rich conditions; when nutrients become limited, reduced mTOR activity permits lipophagic degradation of LDs and supports metabolic adaptation and survival^73–75^. DGAT1 and DGAT2 are essential for LD biogenesis: during transient starvation, they re-esterify free FAs into TAGs, preventing lipotoxicity and ensuring a stable lipid supply that can later be oxidized^5,76^. In polyunsaturated fatty acid (PUFA)-supplemented triple-negative breast cancer cells, inhibiting DGAT-dependent LD biogenesis causes widespread lipidome remodeling that funnels PUFAs from neutral lipids into membrane glycerophospholipids, increasing membrane unsaturation, lipid peroxidation, and ferroptosis sensitivity. LDs normally sequester exogenous PUFAs in TAGs and CEs, but when LD formation is blocked, PUFAs redistribute to ester and ether phospholipids, promoting ferroptosis even without exogenous inducers. In lung adenocarcinoma cells, LDs play a context-dependent role—either protecting against or promoting ferroptosis, depending on PUFA load and FSP1 status—with FSP1 deficiency enhancing LD-linked lipid peroxidation and its propagation, underscoring LDs as multifaceted regulators of ferroptosis vulnerability^77^. Under basal conditions, Perilipin family proteins (PLINs) limit the access of lipolytic enzymes to the LD surface, thereby reducing lipid breakdown and stabilizing LDs. Under starvation, PLINs docked to the phospholipid monolayer upregulate access of lipolytic enzymes to the LD surface. In this process, Comparative Gene Identification-58 (CGI-58), also known as ABHD5 (α/β-hydrolase domain-containing 5), activates ATGL to initiate TAG hydrolysis, generating DAG, which is further hydrolyzed by hormone-sensitive lipase, while monoacylglycerol lipase completes the final step by converting monoacylglycerol into glycerol and free fatty acids. Together, this coordinated lipolytic cascade mobilizes LD-derived FAs for metabolic adaptation under nutrient stress in cancer cells^22,78,79^.

At the mechanistic level, the breakdown of TAGs within LDs is the critical link between energy storage and supply. TAGs are hydrolyzed by lipases such as ATGL, hormone-sensitive lipase, and monoacylglycerol lipase, or degraded via lipophagy, the process that delivers LDs to lysosomes for catabolism^51,80,81^. Access of these lipolytic enzymes to LDs is regulated only in part by PLIN family proteins, which act as gatekeepers of LD lipolysis under basal and stimulated conditions^78^. Liberated FAs are transported into mitochondria through CPT1A and enter β-oxidation, generating acetyl-CoA for the tricarboxylic acid cycle and driving ATP production^82^. This LD-to-mitochondria flux is facilitated by contact sites mediated by PLIN5 and FATP4^83^, and by the activity of peridroplet mitochondria^82,84^, which are specialized to oxidize LD-derived FAs. In glioblastoma and lung cancer models, cells with abundant LDs exhibit superior survival under starvation because they can rapidly mobilize stored FAs, whereas inhibition of LD formation with DGAT inhibitors severely compromises proliferation and survival during nutrient stress^85,86^. Thus, LD breakdown provides critical energy and metabolic flexibility, ensuring cell survival when nutrient supplies are limited. PLIN proteins control the balance of storage and release in healthy tissue, and their upregulation in many cancer types suggests that they control the increased accumulation of LDs in cancer^87–89^. Given that there are multiple PLIN family proteins and lipases, more studies are needed to understand their exact roles in controlling the balance of FA storage and release in LDs in cancer cells.

### LDs in maintaining oxygen homeostasis under hypoxia in cancer

Hypoxia is a defining feature of solid cancers, especially aggressive malignancies^90^. Because cancer growth often outpaces angiogenesis, the central regions of cancers commonly become hypoxic^91^. LD accumulation is consistently observed in hypoxic cancer cells, and hypoxia-related LD remodeling has also been described in colorectal cancer and salivary adenoid cystic carcinoma microenvironments, highlighting a conserved adaptive strategy in certain cancer types^92–95^. Under hypoxic conditions, cancer cells uniformly activate a transcriptional program mediated by hypoxia-inducible factors (HIF-1α and HIF-2α)^96^. These transcription factors drive multiple adaptations that enable cell survival under low oxygen, including lipid metabolic reprogramming. Low oxygen stimulates FA synthesis in breast cancer, with FASN upregulated through the HIF-1α/Akt/SREBP-1 signaling axis^97^. HIF-1α also enhances proliferator-activated receptor gamma expression in peroxisomes, thereby increasing both glycolysis and the uptake of free FAs, collectively promoting LD accumulation^98,99^. Another key adaptive mechanism to hypoxia employed by cancer cells is to buffer lipotoxic stress by adjusting the protein components docked to LDs. HIF-1α upregulates enzymes such as 1-acylglycerol-3-phosphate O-acyltransferase 2 (AGPAT2) in the ER and Lipin-1, which contribute to TAG synthesis at intermediate steps: AGPAT2 promotes phosphatidic acid formation, whereas Lipin-1 converts phosphatidic acid to DAG, the direct precursor of TAG. In this way, excess FAs can be incorporated into TAGs and stored in LDs, thereby protecting cells from lipotoxicity and metabolic instability as demonstrated in hepatoblastoma and cervical adenocarcinoma cells^100,101^. Lipins are intriguing molecules involved in autophagy and inflammation, and their increased expression in cancers suggests they may serve as promising targets for cancer inhibition. Moreover, hypoxia is closely linked to ferroptosis, a non-apoptotic form of cell death driven by iron-dependent lipid peroxidation^93^. Hypoxia can influence ferroptosis susceptibility through several pathways, including changes in iron handling, lipid metabolism, and antioxidant defense, and GPX4 remains a key regulator of lipid peroxide detoxification^94,95^. To counteract this, fibrosarcoma cells activate parallel antioxidant systems: GPX4 reduces toxic lipid peroxides to inert lipid alcohols using glutathione, while ferroptosis suppressor protein (FSP1/AIFM2) regenerates reduced coenzyme Q10 and vitamin K, both of which act as radical-trapping antioxidants^102–104^). In addition, breast cancer cells divert excess PUFAs into TAGs and store them in LDs, effectively buffering oxidative stress from an excess of lipids and sustaining cell viability under the pressure of hypoxia and ferroptosis^105,106^. Together, these findings support an emerging role of LDs as regulators of ferroptosis sensitivity through control of lipid storage, FA unsaturation balance, and oxidative buffering in various cancer types (Table 1).

**Table 1:** Conditions and adaptations in lipid metabolism of cancer cells

Stress Condition	Activated Pathway	Adaptations on LDs	Consequences for Cancer Cells	Reference
Nutrient Starvation	AMPK-Lipophagy	Promotes lysosomal degradation and release of stored Fas	Supports metabolic adaptation and survival during nutrient stress	^68,72^
	AMPK-ACC-CPT1A	Inhibits FA synthesis and promotes FA oxidation	Maintains ATP production and energy homeostasis	^71,73,74^
	mTOR-inhibits lipophagy	Permits lipophagic degradation of LDs	Facilitates catabolic adaptation under nutrient limitation	^75,76^
	DGAT1/2 esterifies FAs into TAGs	Re-esterifies excess FAs into TAGs	Prevents lipotoxicity and preserves viability during transient starvation	^6,78^
	Comparative Gene Identification-58/ATGL-hormone-sensitive lipase-MAGL	Sequential hydrolysis of TAG, DAG, and MAG	Mobilizes stored lipids for fuel use and metabolic flexibility	^80–82^
	Perilipin (PLIN1-5)	Regulate lipases to the LD surface	Regulate LD stability versus lipid mobilization	^49,81^
Hypoxia	HIF-1α/Akt/SREBP-1	Promotes TAG synthesis and storage of excess FAs in LD	Supports growth and adaptation to low oxygen	^100^
	HIF-1α/peroxisome proliferator-activated receptor gamma	Promote glycolysis and uptake of free Fas	Enhances metabolic flexibility and lipid storage	^101,102^
	HIF-1α/Lipin-1 + AGPAT2	Promote TAG synthesis and storage of excess Fas in LD	Buffers lipotoxicity and maintains survival under hypoxia	^103,104^
	GPX4-glutathione	Detoxifies lipid peroxides	Reduces ferroptosis stress and preserves viability	^95,97^
	FSP1/AIFM2-CoQ₁₀/vitamin K	Enhance the radical antioxidant effect	Protects cells from lipid peroxidation and ferroptosis	^105,106^
	PUFA sequestration into TAG	Stores peroxidation-prone PUFAs into LD	Buffers oxidative lipid stress and promotes survival	^107,109^

### LDs in ER homeostasis and proteostasis

LDs originate from the ER, and although they bud into the cytosol, they frequently maintain close associations with the ER through membrane contact sites^107,108^. These connections allow the exchange of lipids, proteins, and signaling molecules. Proteins such as DGAT2 and acyl-CoA synthetases can shuttle between the ER and the LD surface to retain competence for lipid synthesis by the LDs, whereas tethering factors including seipin and Rab18 help stabilize ER–LD contacts and support bidirectional lipid flux^109–111^. Through these connections, excess fatty acids can be re-esterified into TAGs and stored in LDs, while LD-derived lipids can also be mobilized back to the ER when membrane lipid synthesis is needed^111–113^.

This buffering function is particularly important in cancer cells, which commonly increase de novo lipogenesis and accumulate FAs to adapt to their increased demand for nutrients. When saturated FAs such as palmitate build up, the excess lipid saturation alters the transmembrane environment and thereby activates ER stress sensors docked in the ER membrane, including IRE1 and PERK, thereby triggering pro-apoptotic responses^113–119^. By channeling palmitate and other potentially lipotoxic FAs into TAG synthesis and LD storage, cancer cells reduce their accumulation in ER membranes and thereby limit lipotoxic ER stress^120–122^.

**Fig. 2:**
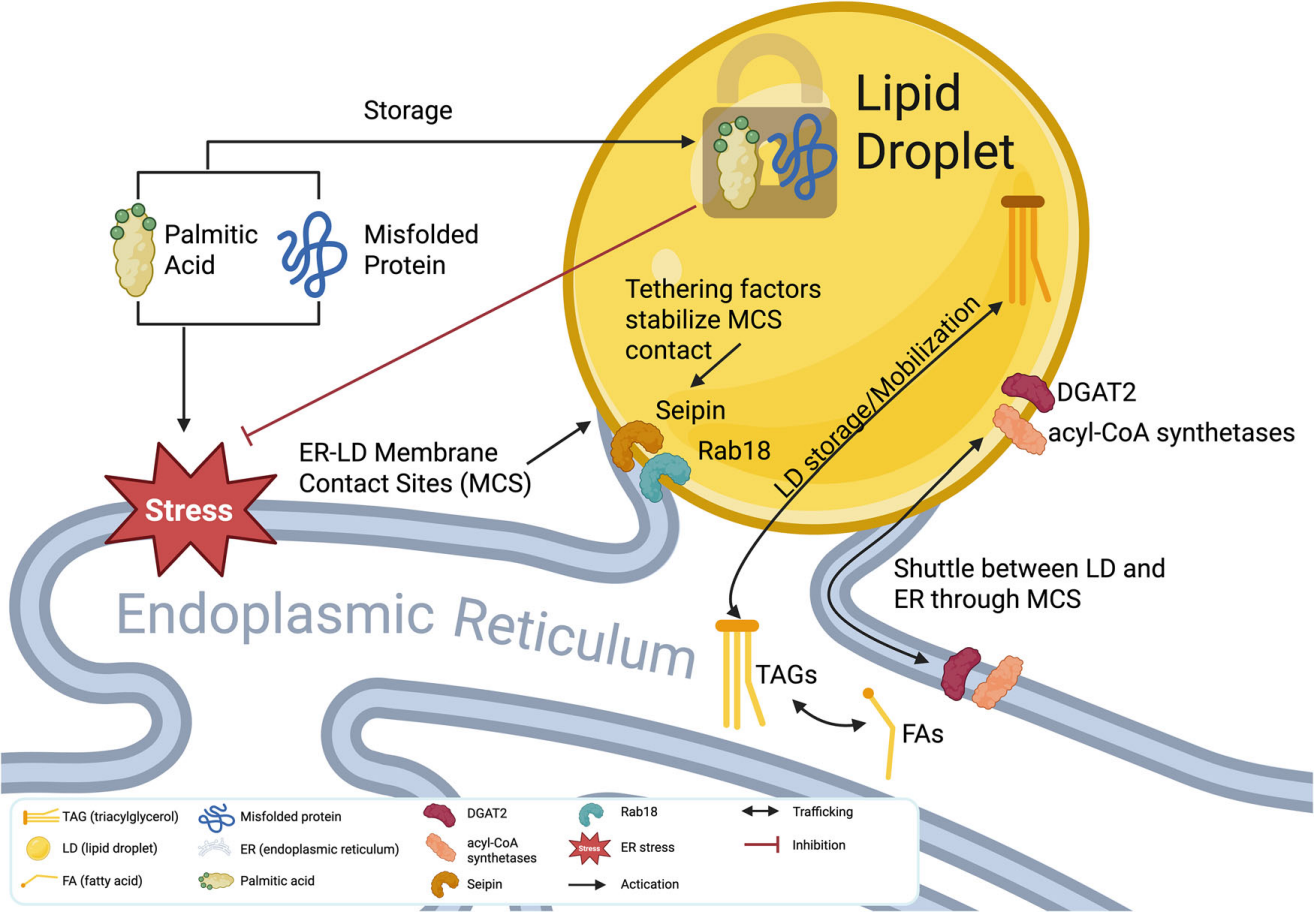
Endoplasmic reticulum–lipid droplet interaction in buffering lipid and proteotoxic stress. Schematic of endoplasmic reticulum (ER)–lipid droplet (LD) membrane contact sites (MCS) and their role in ER homeostasis. LDs bud from the ER, remain connected through membrane contact sites, and exchange lipids and enzymes with the ER. These interactions promote fatty acid (FA) esterification, lipid storage, and clearance of excess or damaged components, thereby helping limit ER stress. LDs may also sequester and isolate lipotoxic species such as palmitic acid, reducing their accumulation in ER membranes and thereby helping prevent ER stress. Additional abbreviations: diacylglycerol O-acyltransferase 2 (DGAT2), acyl-CoA synthetases, and Ras-related protein Rab18 (Rab18). Created with BioRender.com.

LDs may also relieve the proteotoxic burden during ER stress^123^. Under conditions of lipid imbalance or protein misfolding, LDs clear excess lipids and damaged or aggregation-prone proteins by inducing biogenesis and macrolipophagy, the latter requiring direct contact of LDs with the lysosome. This has been linked to restoration of ER homeostasis^124–126^. In cancer, this ER-protective role is likely to support cell survival under chronic metabolic stress and to reduce the unfolded protein response, which is activated as a consequence of ER stress^127^ (Fig. 2).

### LDs in mitochondrial homeostasis under redox stress

LDs have physical and functional contacts with mitochondria through two distinct modes. In the first, consistent with the dynamic interactions observed between other organelles, LDs engage mitochondria via transient “kiss-and-run” contacts, characterized by brief and reversible associations that permit rapid lipid exchange and metabolic signaling^128,129^. In addition, a more stable “anchored” mode is observed between LDs and mitochondria, with the LD-anchored mitochondria tightly bound to LDs even under conditions of mechanical stress^130,131^. These anchored contacts are prevalent in oxidative tissues, including brown adipose tissue, heart, and skeletal muscle, and are thought to provide sustained access to LD-derived FAs, thereby supporting continuous β-oxidation and ATP generation in energy-intensive states^82,131^. In cancer cells, LDs and mitochondria also establish close functional contact, which may support FA transfer, maintain mitochondrial energy production, and increase metabolic flexibility under stress. This interaction is likely to benefit cancer cells by sustaining survival and proliferation when nutrient availability fluctuates or when reliance on FA oxidation increases^132^.

In addition to serving as an energy source, LDs can buffer spikes in FA flux induced by oncogenic signaling in Ras-mutant cancers and by hypoxia, including in breast cancer cells, and prevent mitochondrial stress caused by such sudden surges in fuel delivery. During nutrient deprivation and autophagy, free FAs released from membranes are re-esterified into TAGs by DGAT1 and stored in LDs, thereby preventing a sudden influx of FAs into mitochondria that would otherwise overload the electron transport chain and trigger excessive reactive oxygen species (ROS) production^29,85^.

**Fig. 3:**
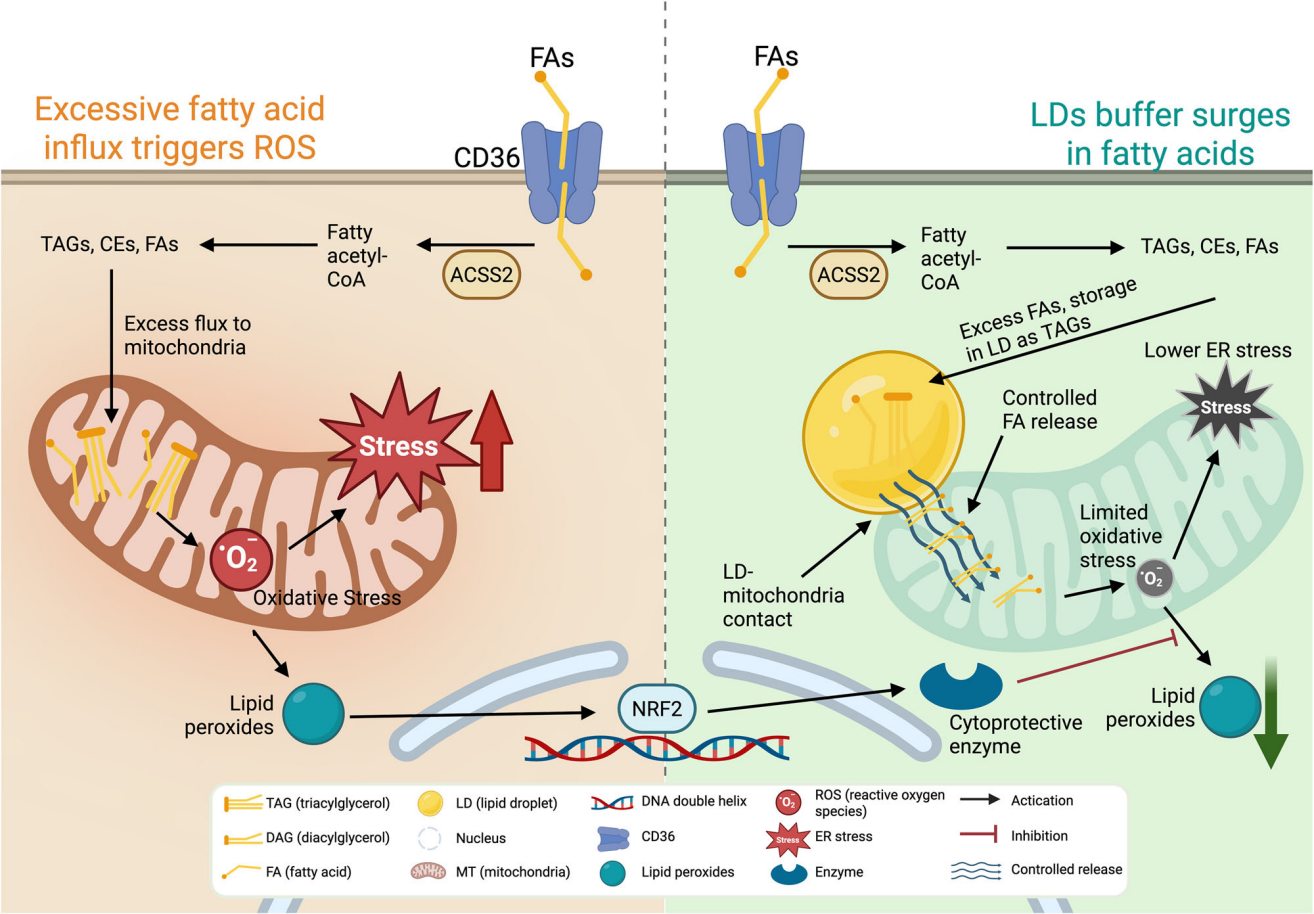
Lipid droplets buffer lipotoxicity through contact with mitochondria. Illustration of how excessive lipid and fatty acid (FA) influx can induce mitochondrial lipotoxicity, reactive oxygen species (ROS) production, lipid peroxide accumulation, and endoplasmic reticulum (ER) stress. Lipid droplets (LDs) buffer this flux by storing excess FAs as triacylglycerols (TAGs), cholesterol esters (CEs), and other lipid species, and through LD–mitochondria contacts they support controlled FA release to mitochondria (MT), thereby limiting oxidative stress. Additional abbreviations: cluster of differentiation 36 (CD36), acetyl-CoA synthetase 2 (ACSS2), fatty acyl-CoA, nuclear factor erythroid 2-related factor 2 (NRF2), diacylglycerol (DAG), and deoxyribonucleic acid (DNA) double helix. Created with BioRender.com.

Subsequently, LDs mobilize FAs in a controlled manner, often through contact sites with mitochondria mediated by proteins such as PLIN5, which enable directed lipid transfer, thereby attenuating oxidative stress while maintaining ATP generation^83,133^. In cancer cells, this buffering function may help preserve mitochondrial fitness, limit lipotoxic and oxidative cell damage, and support continued cancer cell growth under metabolic stress. LD accumulation also intersects with antioxidant signaling because byproducts of lipid oxidation activate the transcription factor Nuclear factor erythroid 2-related factor 2 (NRF2), which induces expression of cytoprotective enzymes and reduces lipid peroxide. The role of NRF2 is complex and cell type-specific, and in a context-dependent manner, NRF2 further enhances cellular redox defenses, which may boost tumor cell survival and therapy resistance under stress conditions^134^ (Fig. 3).

### LDs in nuclear homeostasis and protecting genome integrity

Conventional cancer therapies, which mainly include radiation and chemotherapy, induce DNA damage followed by cell cycle arrest (genotoxic arrest) and cell death in cancer cells^103,135^. In multiple cancer models, including renal cell carcinoma, fibrosarcoma, and melanoma, cell-cycle arrest promotes DGAT1/2 upregulation and DGAT-dependent LD formation, which enhances TAG synthesis, redirects oxidizable FAs into neutral lipid stores, and is associated with increased ferroptosis resistance^103^. To counter therapy-associated stress, cancer cells induce DGAT-dependent LD formation, a protective effect largely lost under lipoprotein-depleted conditions. More generally, exogenous unsaturated FAs can also promote a ferroptosis-resistant state^103,106^. These findings indicate that therapy-induced cell cycle arrest suppresses ferroptosis at least in part by promoting DGAT-mediated TAG synthesis and LD biogenesis. This protective phenotype may depend primarily on the uptake of exogenous lipids. Mechanistically, sequestration of excess FAs into TAG-rich LDs is thought to reduce their incorporation into peroxidation-prone membrane phospholipids and can therefore buffer not only ferroptosis stress but also other forms of lipotoxicity. In this context, by limiting lipid peroxidation and stress-induced cell death during genotoxic arrest, LD formation may help preserve cellular viability and support subsequent recovery of the cell cycle.

The influence of LDs on nucleus homeostasis is, however, not entirely protective. The cell nucleus is one of the stiffest organelles within most cell types. In many cancer cells, such as renal cancer^136^, hepatocellular carcinoma^137^, and breast cancer^138^, LDs become excessively enlarged. With an interfacial tension of around 40 millinewtons/meter, LDs exhibit sufficient mechanical rigidity to deform surrounding cellular structures in osteosarcoma cells. While organelles with more flexible membranes may accommodate such crowding, the stiff nucleus is particularly vulnerable: excessive LD accumulation can physically compress and damage nuclear architecture^139,140^, causing nuclear stress and DNA damage on biophysics level. Molecularly, LDs are mechanically rigid enough to indent the nucleus, dilute Lamin-B1, trigger nuclear envelope rupture, recruit the DNA sensor cGAS, mislocalize DNA repair factors to the cytosol, and consequently increase DNA damage and delay cell cycle progression^140^. Thus, LDs can promote nuclear homeostasis by supporting cell-cycle progression and protecting cells against cell death, including ferroptosis, thereby enhancing cancer cell survival under therapy-induced stress. However, LDs can also function as rigid intracellular stressors that increase the risk of DNA damage.

### LDs as buffers of toxin- and cancer therapy-induced stress

In non-cancer model systems, LDs have been shown to act as transient depots for selected basic, aggregation-prone proteins, including histones. Histones are essential for DNA packaging, yet in excess or in the unchaperoned form, they are highly cytotoxic, as their strong positive charge promotes nonspecific binding to nucleic acids and cellular membranes^141^. For example, nearly 50% of select histones (H2A, H2Av, and H2B) physically associate with LDs. This localization begins during oogenesis and is conserved across species, indicating an evolved buffering function for excess histones^141^. Mechanistically, the LD-resident protein Jabba anchors maternal H2A/H2Av to LDs; when Jabba is disrupted, nuclear H2Av/H2A ratios rise, mitoses falter, and viability drops. Thus, LDs serve not only as lipid depots but also as “histone buffers” and thereby prevent proteotoxicity and maintain nuclear histone balance^142^.

Many chemotherapeutics are highly lipophilic, a property that facilitates membrane permeation but also allows LDs to sequester them, lowering their concentration in the cytosol and attenuating their efficacy. A clear example is provided by FGFR-driven lung cancer cells exposed to the tyrosine kinase inhibitor ponatinib: the inhibitor is selectively taken up by LDs in a process termed “LD-mediated scavenging,” and increased drug LD load correlates with drug resistance, whereas pharmacological inhibition of LD biogenesis (e.g., by Triacsin C) restores sensitivity^143^. Similarly, in acute myeloid leukemia models, resistance to the hydrophobic prodrug CHR2863 is associated with down-regulation of Carboxylesterase 1 (CES1), a critical regulator of lipid mobilization, leading to accumulation of unmetabolized drug in LDs^144^. In hepatocellular carcinoma cells treated with the pan-RAF inhibitor sorafenib, the Aldo-keto reductase family 1 member C3 (AKR1C3) is upregulated, which in turn induces LD accumulation and protects cancer cells from sorafenib-induced mitochondrial lipotoxicity^145^. More broadly, these findings show that by buffering toxic lipids and sequestering selected drugs, the metabolic plasticity provided by LD remodeling supports cancer cell survival under treatment stress and leads to therapy resistance.

### LDs in immune stress adaptation within the tumor microenvironment

**Fig. 4:**
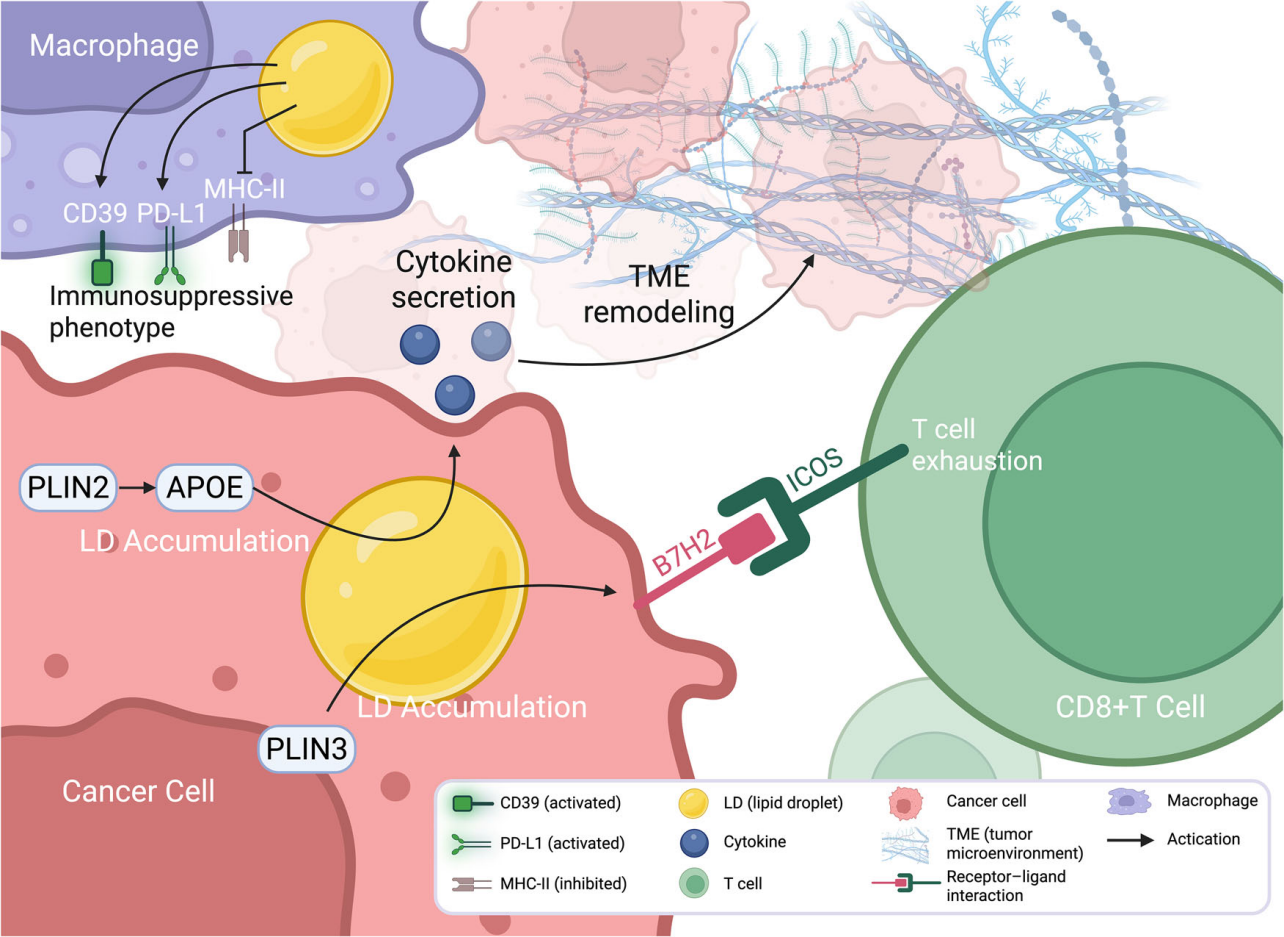
Lipid droplets buffer immune stress and reshape the tumor microenvironment. Schematic illustrating how lipid droplet (LD) accumulation in tumor cells and immune cells contributes to adaptation to immune stress, immune suppression, and stromal remodeling in the tumor microenvironment (TME). In tumor cells, perilipin 3 (PLIN3)-associated LD accumulation is linked to B7 homolog 2 (B7-H2)-related signaling and CD8-positive T-cell exhaustion, whereas perilipin 2 (PLIN2)/apolipoprotein E (APOE)-driven LD formation promotes cytokine secretion and extracellular matrix stromalization. In macrophages, LD-rich states are associated with increased cluster of differentiation 39 (CD39) and programmed death-ligand 1 (PD-L1) and reduced major histocompatibility complex class II (MHC-II) expression, consistent with an immunosuppressive phenotype. Additional abbreviations: inducible T-cell co-stimulator (ICOS), cytotoxic T lymphocyte marker CD8 (CD8), and T cell. Created with BioRender.com.

The role of LDs in maintaining tumor cell homeostasis is not limited to cancer cell-intrinsic mechanisms but extends to the tumor microenvironment. In this context, LDs may help tumors evade immune surveillance and escape the anti-tumor immune response. In oral squamous cell carcinoma, PLIN3-high tumor cells showed increased LD deposition and were associated with reduced CD8 + T-cell activation and greater T-cell exhaustion, partly through B7-homolog 2-related signaling (Fig. 4)^146^. In esophageal squamous cell carcinoma, PLIN2/APOE-driven LD formation promotes cytokine secretion, stromalization, and suppression of T-cell activation^147^. Moreover, beyond their effects in tumor cells, LDs can also accumulate directly within immune cells and thereby contribute to immune evasion. In macrophages, LD-rich states have been linked to immunosuppressive phenotypes and tumor-promoting functions. For example, LD–loaded macrophages have been identified in human glioblastoma and were associated with an immunosuppressive state marked by increased CD39 and PD-L1 and reduced Major Histocompatibility Complex Class-II expression, suggesting that LD accumulation may support macrophage adaptation to the hostile tumor milieu while weakening anti-tumor immunity^148^. Interestingly, LDs actively participate in mammalian innate immunity by both cell-autonomously killing intracellular pathogens and locally, and by facilitating systemic metabolic adaptation to infection. Together, these findings suggest that LDs may buffer metabolic and oxidative stress not only in cancer cells but also across tumor–immune interactions, thereby reinforcing immune evasion and escape (Fig. 4). Further research is needed to illuminate the complex roles for LDs in modulating the anti-cancer immune response. Moreover, we are only beginning to understand their potential interference with emerging immunotherapies^149^.

### Experimental approaches for studying lipid droplets

Fluorescence-based imaging remains the most widely used method to detect and quantify LDs in cells. Fluorescent dyes such as BODIPY and Nile Red are convenient tools for visualizing LD abundance, size, and intracellular distribution, and combined with widefield or confocal microscopy and imaging software, they can be used for quantitative analysis^150,151^. However, these dyes do not provide a complete biochemical readout of LD composition, and their specificity is not absolute, especially under conditions with abundant neutral lipids or in the presence of other hydrophobic intracellular structures^152,153^. Ultrastructural methods, including electron microscopy and correlative light-electron microscopy, provide much higher spatial resolution and are particularly valuable for examining LD interactions with the ER, mitochondria, and other organelles, although they are lower-throughput and technically demanding^154^.

Label-free approaches such as Raman spectroscopy and stimulated Raman scattering imaging add an important layer of visualizing lipid-rich structures without exogenous dyes and provide information on lipid composition and saturation state^155,156^. But these methods generally infer chemical signatures rather than directly proving organelle identity unless combined with imaging or marker-based validation^156^. Biochemical approaches, including LD isolation followed by analyzing their contents with proteomic or lipidomic approaches, are useful for identifying cell-type-specific and context-dependent proteins and lipid species associated with LDs^157,158^. Additionally, stable isotope tracing can reveal lipid flux into and out of LDs^159^. Still, biochemical profiling alone cannot fully exclude contamination from close contact organelles and therefore requires independent confirmation^157^. Finally, functional assays such as lipolysis measurements, lipophagy assays, and organelle contact-site analysis are essential for linking LD morphology to its function in controlling metabolic activity and stress adaptation^160–162^. Together, each method mentioned above (Table 2) reveals a specific aspect of LD biology; it is generally best to combine at least two complementary approaches when studying LDs' morphology, composition, and function.

**Table 2:** Common experimental approaches for studying lipid droplets

Approach category	Methods	Major Strength	Limits
Fluorescence imaging	BODIPY/Nile Red under widefield microscopy/confocal microscopy	Simple and widely accessible for visualizing LD abundance, size, and number	Dye specificity is not absolute; it does not directly define LD biochemical composition
Ultrastructural imaging	Electron microscopy (EM), correlative light-electron microscopy (CLEM)	High spatial resolution shows ultrastructure and interactions with other organelles	Low-throughput; technically demanding; sample preparation is time-consuming
Label-free vibrational imaging	Raman spectroscopy, stimulated Raman scattering (SRS), coherent Raman scattering (CRS)	Label-free detection of lipid-rich structures provides in situ information on lipid composition	Infer chemical signatures rather than proving organelle identity, an indirect measure of LD composition
Biochemical profiling	LD isolation, LD proteomics, LD lipidomics	Identifying LD-associated proteins and LD molecular composition	Imprecise, cannot fully exclude compounds from closely associated membranes
Metabolic flux profiling	Stable isotope tracing	Provides dynamic info on lipid uptake, synthesis, storage, mobilization, and oxidation	Interpretation depends on tracer design and experimental context; needs combined with imaging assays to localize the flux site
Functional assays	Lipolysis measurements, lipophagy assays	Useful for showing lipid mobilization, catabolism, organelle coupling, metabolic activity, and stress adaptation	Captures only one aspect of lipid biology at a time, and needs to be combined with imaging assays to localize the functional site

## Outlook

In recent years, researchers have increasingly recognized lipid LD accumulation as a prevalent phenotype across cancers, especially in highly malignant, recurrent, and therapy-resistant cancers. Our understanding has shifted from viewing LDs as inert energy depots to recognizing them as dynamic organelles that maintain cancer cell homeostasis and enable stress adaptation. Through crosstalk with other organelles and active participation in energy and redox metabolism, LDs help stabilize the intracellular milieu when cancer cells are instructed to proliferate and progress by oncogenic signaling, and in the process encounter complex external pressures. In addition to the organelles discussed here, LDs may also interact with other compartments such as peroxisomes, but current evidence remains limited and their significance in cancer awaits further study^163^. The field of cancer lipid metabolism and LD biology thus stands at a crossroads of pressing challenges and promising opportunities.

Despite these promising LD-directed strategies across multiple experimental systems, several issues must be addressed before broad clinical translation, foremost among them, the therapeutic specificity of targeting LD in cancer. Unlike cancer-restricted antigens exploited by immunotherapies, the machinery supporting LD biogenesis and turnover is widely present in normal tissues. As a result, systemic LD-targeting strategies may cause severe adverse effects, including metabolic imbalance and weight loss, as suggested by preclinical studies^164,165^. These adverse effects need to be addressed, especially since patients with low baseline weight or poor nutritional status often have worse outcomes clinically. Developing metabolism-targeting agents with increased specificity is critical to minimize collateral metabolic perturbation.

A second major direction for future preclinical research is to define which aspects of LD biology are most therapeutically actionable. This issue should be considered in the context of existing preclinical studies targeting LD biogenesis and other LD-related pathways, rather than focusing only on upstream de novo fatty acid synthesis. Notably, the DGAT pathway, which directly participates in neutral lipid synthesis and LD formation, has already been explored therapeutically in non-oncology settings, supporting the druggability of this axis^166,167^. At the same time, translating this strategy into cancer will require careful attention to tumor specificity and treatment tolerability. Several important molecular questions remain open, including identifying tumors that strongly depend on TAG-rich versus CE-rich LD pools, which specific LD-associated proteins may be more selective drug targets than broader lipid synthesis pathways, and how inhibition of LD formation, turnover, or stress-buffering function can be most effectively combined with radiotherapy, ferroptosis-inducing strategies, targeted therapy, or immunotherapy.

Several biological questions also remain open. One is the extent of LD heterogeneity across cancer types and states, including differences in LD size, composition, and associated protein cargo. Another is the need to better define LD-mediated drug resistance mechanisms, including whether LDs mainly act through sequestration of lipophilic drugs, buffering of toxic lipids, or broader metabolic rewiring during treatment adaptation. A third unresolved issue is the role of LDs in ferroptosis and oxidative lipid metabolism, including how different tumors balance PUFA storage, MUFA availability, lipid peroxidation, and antioxidant systems under stress. Fourth, the roles for LDs in cell fate changes, including epithelial-mesenchymal transitions and malignant transformation, remain largely unexplored. In parallel, an important direction is to clarify how LD remodeling in cancer cells and immune cells, especially macrophages, dendritic cells, and exhausted T cells, shapes antigen presentation, inflammatory signaling, stromal remodeling, and immune suppression within the tumor microenvironment.

Together, these questions suggest that the next stage of the field should move beyond describing LD accumulation as a cancer phenotype and instead define when, where, and how LDs become true therapeutic liabilities. A better understanding of LD heterogeneity, LD-dependent stress adaptation, and tumor-specific metabolic context will be essential for translating LD biology into more precise and effective cancer treatment strategies.

### Reporting summary

Further information on research design is available in the Nature Portfolio Reporting Summary linked to this article.

## Supplementary information

Transparent Peer Review file

Reporting Summary

## Data Availability

N/A
