# Supplementary material for: Lipid droplets as stress-buffering organelles in cancer cell homeostasis
Source: Commun Biol. 2026 Jun 29;9:868. doi: 10.1038/s42003-026-10566-5 (PMC13315355; doi:10.1038/s42003-026-10566-5)
Supplement: Supplementary file 1 — Transparent Peer Review file [file 42003_2026_10566_MOESM1_ESM.pdf]

nature portfolio

Peer Review File

Lipid Droplets as Stress-Buffering Organelles in Cancer Cell Homeostasis

Corresponding Author: Professor Claudia Petritsch

This file contains all reviewer reports in order by version, followed by all author rebuttals in order by version.

Version 0:

Reviewer comments:

Reviewer #1

(Remarks to the Author)
Authors have explored the significance of lipid droplets (LDs) in cancer, focusing on their biosynthesis, composition, and their roles in maintaining energy and oxygen cell homeostasis. They also examined the interactions of LDs with the endoplasmic reticulum, mitochondria, and the nucleus, as well as the subsequent effects related to toxin management. While the manuscript is well-articulated, it would benefit from language editing. The authors' approach to this subject is both intriguing and innovative; however, the discussion lacks depth, which could enhance the reader's understanding by broadening the scope beyond a few specific cancer examples.
Here are some suggestions:
• The introduction fails to adequately set the stage for the subsequent paragraphs, particularly regarding the review's discussion of LDs.
• The graphical abstract does not accurately reflect the review's content.
• The organization of the paragraphs is somewhat misleading, beginning with the formation of LDs, followed by composition, energy, and oxygen homeostasis. However, the subsequent sections shift focus to organelles rather than maintaining a consistent emphasis on relevant factors. Authors are encouraged to revise their paragraph headings to ensure greater coherence throughout the text.
• In Figure 1:
1. Authors need to maintain consistency in their representation of stimuli, pathways, and the enzymes that are activated or repressed; for instance, the stimulus related to PI3K is unclear.
2. Furthermore, the schematic representation of fatty acids requires revision, as it does not accurately depict a fatty acid.
3. The term "cancer-specific LDs assembly" needs clarification—do the authors mean accumulation or formation?
4. The legend currently highlights only some oncogenic pathways, leaving out others.
• What do authors mean by the “molecular” composition of LDs.
• Line 86 lacks a reference and does not specify the type of remodelling being discussed, whether it pertains to lipids or proteins, necessitating further detail.
• It should be noted that the hydrophobic core can also contain occasionally vitamins and other hydrophobic compounds.
• Line 92 is missing a reference.
• In Line 94, the authors should elaborate on how oncogenic signalling impacts cancer cells.
• Line 112, why authors only mentioned ATGL and no other lipolytic enzymes, with more details to be included in that discussion.
• In Table 1, it would be beneficial to add a column addressing the effects/consequences on cancer cells, along with another column for references.
• The paragraph on ER homeostasis lacks clarity regarding how LDs contribute to preventing ER stress and their role in tumorigenesis.
• In Figure 2, the proteins identified as contact sites should be shown to physically connect LDs to the endoplasmic reticulum.
• The paragraph on mitochondrial homeostasis also requires additional details about its consequences for cancer cells.
• The schematic in Figure 3 is very simple and would benefit from more intricate details.

Reviewer #2

(Remarks to the Author)
This manuscript reviews the role of lipid droplets (LDs) in maintaining cancer cell homeostasis under diverse cellular stresses including nutrient deprivation, hypoxia, ER stress, mitochondrial dysfunction, and exposure to toxic compounds. The authors propose that LDs act as central hubs coordinating metabolic buffering, lipid detoxification, and inter-organelle communication, thereby enabling tumor cell survival and therapy resistance.
The manuscript is generally well organized and readable, and the conceptual framework linking LD biology to cellular homeostasis is a useful perspective. However, the novelty relative to recent literature is moderate, several sections rely on broad generalizations rather than clearly distinguishing established mechanisms from emerging hypotheses, and the manuscript would benefit from additional recent references and a clearer discussion of experimental methodologies used to study lipid droplets.
Overall the review could become a strong contribution with revisions that sharpen the conceptual positioning and expand key sections.

Major Comments
1. Novelty relative to recent reviews
The manuscript covers many aspects already addressed in recent reviews on lipid droplets in cancer and LD-organelle interactions. For example:
• Cancer-focused LD biology reviews published in 2023–2025
• Reviews on LD–organelle crosstalk
• Reviews discussing LD heterogeneity and lipid metabolism in cancer
While the current manuscript frames LDs as mediators of cellular homeostasis, the overlap with these publications is substantial. The authors should explicitly state how their review differs from existing literature and clarify what new conceptual framework it provides.
For example, the manuscript could position itself as a review focused specifically on:
• LDs as stress buffering organelles
• LD-mediated metabolic resilience mechanisms
• LDs linking lipid metabolism and proteotoxic stress
Without this clarification the manuscript risks appearing redundant.

2. Insufficient coverage of recent literature
Although the manuscript includes foundational studies, several sections rely heavily on older references and should incorporate more recent work.
In particular, the following areas require updated citations:
• LD-organelle contact sites
• LD heterogeneity and lipid composition
• LDs in immune cells within the tumor microenvironment
• LDs in therapy resistance and metabolic plasticity
• Methods for studying LD biology
Recent reviews and studies published in the last 3–5 years should be incorporated to ensure the review reflects the current state of the field.

3. Overgeneralization of mechanistic conclusions
Several sections present findings from specific systems as broadly applicable mechanisms across cancer types.
Examples include:
• hypoxia-mediated LD accumulation
• GPX4 activity and ferroptosis regulation
• sequestration of chemotherapeutic agents in LDs
• lipid droplet–mediated detoxification
In many cases these mechanisms have been demonstrated only in particular tumor models or cell lines. The manuscript should clarify when observations are context-specific rather than universal features of cancer biology.

4. Mechanistic inaccuracies or imprecise wording
A few statements would benefit from correction or clarification.
Examples:
COX-2 description
The manuscript states that COX-2 catalyzes the synthesis of arachidonic acid into prostaglandins. COX-2 instead converts arachidonic acid into prostaglandin endoperoxides (PGG2/PGH2), which are then converted into prostaglandins by downstream enzymes.
FIT2 gene nomenclature
The gene is generally referred to as FITM2, not FIT2M.
Lipid droplet growth mechanisms
The manuscript presents coalescence and Ostwald ripening as definitive explanations of LD growth. These processes are plausible biophysical mechanisms but should be described as models rather than universal mechanisms in living cells.
GPX4 regulation
The statement that oxygen levels directly modulate GPX4 activity is mechanistically vague and should be clarified. Hypoxia influences ferroptosis susceptibility through several pathways, not necessarily direct modulation of GPX4 enzymatic activity.

5. Missing discussion of experimental methodologies
The manuscript would greatly benefit from a short section describing how lipid droplets are studied experimentally. This

addition would increase the utility of the review for readers entering the field.
Possible subsections could include:
Imaging approaches
• BODIPY / Nile Red staining
• confocal microscopy
• electron microscopy
• correlative light-electron microscopy
Label-free methods
• Raman spectroscopy
• stimulated Raman scattering imaging
Biochemical approaches
• lipid droplet isolation
• LD proteomics and lipidomics
• isotope tracing for lipid flux
Functional assays
• lipolysis assays
• lipophagy measurements
• organelle contact site analysis
Such a section would make the review significantly more informative.

6. Tumor microenvironment and immune cells
The role of lipid droplets in immune cells (e.g., tumor-associated macrophages, dendritic cells, T cell exhaustion) is mentioned only briefly in the future directions section.
However, LD metabolism in immune cells is now recognized as an important aspect of tumor biology and therapy response. Expanding this discussion would strengthen the translational relevance of the review.

Minor Comments
1. Several sections could benefit from clearer transitions to improve narrative flow.
2. The manuscript should distinguish between neutral lipid storage (TAG vs cholesteryl esters) and their distinct roles in cancer metabolism.
3. The future directions section is somewhat generic. It could be improved by highlighting specific unresolved questions, such as:
o LD heterogeneity across cancer types
o lipid droplet–mediated drug resistance mechanisms
o LD involvement in ferroptosis and oxidative lipid metabolism
4. Figures illustrating LD-organelle interactions or metabolic pathways would improve clarity.

Suggested Additional Topics
The manuscript would benefit from including discussion of:
• lipid droplet heterogeneity
• spatial lipidomics and LD composition
• LD–peroxisome interactions
• LD regulation of ferroptosis
• lipid droplets in metastatic cancer cells
• metabolic plasticity during therapy resistance

Overall Recommendation
The manuscript provides a useful overview of lipid droplets as regulators of cancer cell homeostasis. However, revisions are required to strengthen its novelty, update the literature coverage, clarify mechanistic claims, and include methodological perspectives.
With these improvements the review could become a valuable contribution to the field.

Reviewer #3

(Remarks to the Author)
In this review paper, the authors discuss the expanding roles of lipid droplet as multifaceted organelles in cancer. The topic is interesting and timely. The manuscript is generally well structured, and the categorization of LD functions helps guide the reader through the diverse roles attributed to these organelles. However, several sections are quite dense, presenting substantial information with limited context. More importantly, the discussion too often stays on the surface, collecting existing data well, but without providing much in-depth mechanistic knowledge or novel concepts. In addition, there are several instances with poor explanation and even errors in general lipid biochemistry, which is surprising, as this should be fundamental for a review centred on a lipid storage and trafficking organelle, which discusses functions related to fatty acid trafficking, membrane homeostasis, energy production, ER stress, etc. Finally, the figures are too general, they show limited mechanistic detail. More importantly, the information provided in the figures is not clearly presented and is (too) often unclear and even wrong. In my humble opinion, a substantial revision would be required to enhance this manuscript to the level expected by the readers of Comms Biology.

Major:
1. Explanation of the key biochemical pathways leading to neutral lipid synthesis would be very beneficial for the paper, as many of the enzymes are mentioned in the review but their role in lipid and LD metabolism is not explained.
2. Figure 1: The lines/pathways connecting the various enzymes and elements are not clear. How are DGAT1/2 and ACLY directly linked? Why does FA uptake through CD36 proceed via the nucleus? “Lipids and FAs” are produced by FASN? Are the lipids depicted as small droplets somehow involved in generating bigger droplets?
3. The chapter on the “molecular composition of LDs” should be revised/expanded to clarify and specify in more detail how is the lipid/protein composition of LDs altered in cancer. For example, it is not clear from the text how and which changes in DGAT1/2 are cancer-specific? The relevance of the findings that eicosanoids are present at the surface of LDs should be clarified. Does eicosanoid production occur at the LD surface or at the ER membrane nearby? Which LD enzymes are involved? Are these oxylipins released from cells? Is this mechanism cancer-specific?
4. Line 107: Could the authors provide a reference that links mTOR with lipophagy and not lipolysis?
5. The authors should clarify how lipin-1 and AGPAT2 “catalyze TAG synthesis”?
6. Lines 144-146: This section should be clarified. Could the authors provide more details on how cancer cells divert PUFAs into LDs? The referenced study by Magtanong et al only addressed the role of exogenous MUFAs in ferroptosis, but there is indeed ample evidence that PUFA storage is protective against ferroptosis, but also other forms of lipotoxicity.
7. Table 1 is interesting but should provide key references for each statement.
8. Fig. 2: The figure should be thoroughly revised as the graphical elements are misleading and not clear. Why is a TAG molecule depicted near Rab18? Which MCS sites are depicted? Does palmitic acid block ER stress as suggested? Is there a shuttle from the ER to LDs?
9. Fig legends should contain short descriptions of the depicted pathways and mechanisms.
10. Fig. 3. TG molecule is labeled as FA. Which is the complex lipid presented on top, containing 5 FA chains and two headgrops? Are TGs molecules transported through channels on the plasma membrane?
11. Lines 212-216: Revise to clarify how is LD PUFA storage mechanistically linked to nuclear homeostasis?
12. Line 222: Are LDs biomolecular condensates?
13. Line 261: The prospect of targeting LDs for therapy should be discussed in the context of numerous existing preclinical studies using DGAT inhibitors in mice or humans, or other LD targets, and not FASN inhibitors, which inhibit de novo FA synthesis and do not block LD metabolism per se.

Minor:
Abbreviations are not defined. And when defined they are not consistently used. And some abbreviations are defined multiple times.
All abbr. in figures should be defined.
Fig. 2: what is Acyl-Coa syn?

Version 1:

Reviewer comments:

Reviewer #1

(Remarks to the Author)
The authors have satisfactorily addressed most of my concerns in the revised version of the manuscript. The narrative flow has been improved, with a particular emphasis on specific types of cancer being highlighted. Additionally, the overall quality of the manuscript has been improved. Therefore, I have no additional comments to provide.

Reviewer #3

(Remarks to the Author)
The authors have significantly improved the paper and addressed the critical issues. The paper reads much better and provides a balanced view on current research while identifying some of the critical open questions.

However, some of the details in the figures still reveal significant inaccuracies in the understanding of lipid metabolism and cell biology.
For example, in several figures ACSS2 is depicted as acting on fatty acids (and activating them??) taken up through CD36. As far as I know, this enzyme is very specific for acetate, driving de novo lipogenesis... The whole downstream pathway leading to LD accumulation in these figures therefore does not make any sense.

Another example - TGs are depicted with a benzene ring and 5 acyl chains... Cholesterol also contains benzene and 2 chains apparently? In figure 1, these TGs, CEs are depicted in the cytosol and away from lipid droplets, which are shown a bit lower. Why?

Open Access This Peer Review File is licensed under a Creative Commons Attribution 4.0 International License, which permits use, sharing, adaptation, distribution and reproduction in any medium or format, as long as you give appropriate credit to the original author(s) and the source, provide a link to the Creative Commons license, and indicate if changes were made.
In cases where reviewers are anonymous, credit should be given to 'Anonymous Referee' and the source.
The images or other third party material in this Peer Review File are included in the article's Creative Commons license, unless indicated otherwise in a credit line to the material. If material is not included in the article's Creative Commons license and your intended use is not permitted by statutory regulation or exceeds the permitted use, you will need to obtain permission directly from the copyright holder.
To view a copy of this license, visit https://creativecommons.org/licenses/by/4.0/

Dear Editor Georgieva and Reviewers,

We sincerely thank you for your careful evaluation of our manuscript and for the constructive and insightful comments. These suggestions have been highly valuable in improving the clarity, rigor, and overall quality of our review.

In response to the comments, we have made major and substantive revisions throughout the manuscript. Specifically, we revised wording and phrasing across multiple sections to improve clarity, accuracy, and narrative flow. We substantially redesigned the original Graphic Abstract and redrew Figures 1–3 to better reflect the updated structure and central concepts of the review. In addition, we introduced two new paragraphs, one new figure, and one new table to improve the manuscript's completeness and readability. We believe that these revisions have significantly strengthened the manuscript.

Below, we provide a point-by-point response to all comments. Reviewer comments are shown in **bold**, and our responses are shown in *italics*.

Reviewers' comments:

Reviewer #1 (Remarks to the Author):

Comment 1.1. The introduction fails to adequately set the stage for the subsequent paragraphs, particularly regarding the review's discussion of LDs.

Re: We substantially revised the Introduction to better set the stage for the rest of the review. In the revised version, the Introduction now more clearly reflects the overall structure and central concept of the manuscript, particularly the framing of LDs as stress-buffering organelles that support cancer cell homeostasis under multiple forms of stress. We believe this rewritten Introduction now provides a stronger conceptual foundation for the subsequent sections.

Comment 1.2. The graphical abstract does not accurately reflect the review's content.

Re: We have substantially redesigned the graphical abstract to better match the updated manuscript. The revised version now more clearly summarizes both aspects of LD biology emphasized in the review: the fields in the top half outlines LD interactions with organelles and the microenvironment, while the fields in the bottom half outlines the corresponding

stress-buffering functions of LDs in cancer. We believe this revised graphical abstract now reflects the overall structure, central concept, and updated content of the review more accurately.

Comment 1.3. The organization of the paragraphs is somewhat misleading, beginning with the formation of LDs, followed by composition, energy, and oxygen homeostasis. However, the subsequent sections shift focus to organelles rather than maintaining a consistent emphasis on relevant factors. Authors are encouraged to revise their paragraph headings to ensure greater coherence throughout the text.

Re: Thank you for this suggestion. Paragraph titles have been updated for better consistency, and all titles now reflect that LDs support different dimensions of homeostasis.

Comment 1.4. In Figure 1,

Comment 1.4.1. Authors need to maintain consistency in their representation of stimuli, pathways, and the enzymes that are activated or repressed; for instance, the stimulus related to PI3K is unclear.

Re: The upstream trigger for the PI3K pathway has been clarified in the revised figure. We now indicate annotation 'oncogenic' of PI3K to better reflect cancer-associated activation of the PI3K–AKT–mTOR–SREBP lipogenic axis.

Comment 1.4.2. Furthermore, the schematic representation of fatty acids requires revision, as it does not accurately depict a fatty acid.

Re: The schematic symbol for FAs has been updated. It now shows both saturated and unsaturated fatty acids, with a long zig-zag hydrocarbon chain and a carboxyl head group.

Comment 1.4.3. The term "cancer-specific LDs assembly" needs clarification—do the authors mean accumulation or formation?

Re: The title has been updated to "Cancer-associated lipid droplet formation and accumulation," because the figure shows not only lipid droplet synthesis, but also related pathways that promote lipid droplet formation and accumulation in cancer cells.

Comment 1.4.4. The legend currently highlights only some oncogenic pathways, leaving out others.

Re: The legend has been further expanded to include additional related pathways that were not clearly described before. In the revised version, we now also include the LPS/Inflammation/TLR4/LPCAts pathway, the synthesis of neutral lipids from fatty acids, the formation of small lipid droplets from neutral lipids, the Ostwald ripening of small lipid

droplets into larger droplets and related regulators such as seipin, and lipid droplet degradation.

Comment 1.5. What do authors mean by the "molecular" composition of LDs.

Re: We thank the reviewer for the opportunity to make a correction. We agree that the previous title was not fully accurate. Since this section discusses both the changing composition of LDs and their functional relevance in cancer cells, the title has been revised to "Structural and functional remodeling of LDs in cancer".

Comment 1.6. Line 86 lacks a reference and does not specify the type of remodelling being discussed, whether it pertains to lipids or proteins, necessitating further detail.

Re: We have revised this section to clarify that the remodeling discussed here refers to both changes in LD-associated protein cargo and broader lipid metabolic rewiring in cancer cells, and we added supporting references (Accioly MT, Pacheco P, Maya-Monteiro CM, Carrossini N, Robbs BK, Oliveira SS, et al. Lipid Bodies Are Reservoirs of Cyclooxygenase-2 and Sites of Prostaglandin-E2 Synthesis in Colon Cancer Cells. Cancer Res. 2008 Mar 13;68(6):1732–40. doi:10.1158/0008-5472.CAN-07-1999).

Comment 1.7. It should be noted that the hydrophobic core can also contain occasionally vitamins and other hydrophobic compounds.

Re: Thank you for this helpful suggestion. We agree with this point, and we have now added this sentence together with the corresponding reference (Imanishi Y, Gerke V, Palczewski K. Retinosomes : new insights into intracellular managing of hydrophobic substances in lipid bodies. J Cell Biol. 2004 Aug 16;166(4):447–53. doi:10.1083/jcb.200405110).

Comment 1.8. Line 92 is missing a reference.

Re: Thank you for this helpful comment. You are right that the supporting reference was missing in this sentence. We have now added the relevant references showing that AMPK is activated when cellular energy is low and that AMPK regulates lipid droplet mobilization under starvation conditions (Hawley SA, Davison M, Woods A, Davies SP, Beri RK, Carling D, et al. Characterization of the AMP-activated protein kinase kinase from rat liver and identification of threonine 172 as the major site at which it phosphorylates AMP-activated protein kinase. J Biol Chem. 1996 Nov 1;271(44):27879–87. doi:10.1074/jbc.271.44.27879 PubMed PMID: 8910387.; Singh R, Kaushik S, Wang Y, Xiang Y, Novak I, Komatsu M, et al. Autophagy regulates lipid metabolism. Nature. 2009 Apr 30;458(7242):1131–5. doi:10.1038/nature07976 PubMed PMID: 19339967; PubMed Central PMCID: PMC2676208.).

Comment 1.9. In Line 94, the authors should elaborate on how oncogenic signalling impacts cancer cells.

Re: We have revised this part to better describe how oncogenic signaling impacts this process, particularly that PI3K–AKT–mTOR signaling promotes anabolic lipid metabolism and suppresses autophagy under nutrient-rich conditions. In contrast, nutrient stress reduces mTOR activity and allows AMPK-associated lipophagic LD mobilization to support metabolic adaptation and survival.

Comment 1.10. Line 112, why authors only mentioned ATGL and no other lipolytic enzymes, with more details to be included in that discussion.

Re: We agree that mentioning only ATGL was too limited. We have revised this part to include the sequential roles of other lipolytic enzymes aside from ATGL, including HSL, and MAGL, in LD lipolysis, We also clarified that perilipin proteins regulate the access of these lipolytic enzymes to the LD surface, with CGI-58 serving as a cofactor for ATGL activation.

Comment 1.11. In Table 1, it would be beneficial to add a column addressing the effects/consequences on cancer cells, along with another column for references.

Re: Thank you for this helpful suggestion. We have redesigned Table 1 by adding one column describing the consequences for cancer cells and another column listing references for each statement. At the same time, we also revised the "Adaptations on LDs" column to make the wording more precise and rigorous.

Comment 1.12. The paragraph on ER homeostasis lacks clarity regarding how LDs contribute to preventing ER stress and their role in tumorigenesis.

Re: We have revised that paragraph to better clarify that ER-associated LDs help buffer lipotoxic and proteotoxic stress by sequestering excess fatty acids into TAGs, reducing their accumulation in ER membranes, and helping relieve the burden of lipid and protein imbalance in cancer. We also now more explicitly state that this ER-protective function may support cancer cell survival under chronic metabolic stress and thereby contribute to tumor progression.

Comment 1.13. In Figure 2, the proteins identified as contact sites should be shown to physically connect LDs to the endoplasmic reticulum.

Re: We agree with the reviewer that Figure 2 needed some clarification. We have therefore redrawn. In the revised manuscript, we depict ER–LD interaction including the membrane contact sites (MCSs) and the proteins involved in these contact sites. We depict the dynamic movement of DGAT2 and acyl-CoA synthetases through MCSs along the ER–LD interface. In addition, we corrected the previous presentation of palmitic acid and UPR and

related ER stress pathway. The revised figure now better illustrates that lipotoxic fatty acids such as palmitate may be esterified and stored in LDs, thereby reducing their accumulation in ER membranes and helping to limit ER stress, rather than directly suggesting that palmitic acid itself blocks ER stress.

Comment 1.14. The paragraph on mitochondrial homeostasis also requires additional details about its consequences for cancer cells.

Re: We have revised this section to more clearly state the effects of mitochondrial homeostasis in glioma, osteosarcoma and other cancer cells. Specifically, we describe that LD–mitochondria coupling promotes cancer cell survival, growth, and stress tolerance by supporting fatty acid transfer, maintaining mitochondrial energy production, increasing metabolic flexibility, and limiting lipotoxic and oxidative damage.

Comment 1.15. The schematic in Figure 3 is very simple and would benefit from more intricate details.

Re: Thank you for the suggestion. We have thoroughly revised this figure. In the revised Figure 3, we outline the dynamics of free fatty acids, TAG storage within LDs, and fatty acid transfer to mitochondria. We removed the misleading presentation suggesting that TG is transported through plasma membrane channels and corrected the lipid structures shown in the schematic. We also added more mechanistic detail to illustrate that excess fatty acids can be re-esterified and stored in LDs, followed by controlled transfer to mitochondria through LD–mitochondria contact, thereby limiting ROS accumulation and preserving mitochondrial function.

Reviewer #2 (Remarks to the Author):

Major Comments

Comment 2.1. Novelty relative to recent reviews

The manuscript covers many aspects already addressed in recent reviews on lipid droplets in cancer and LD-organelle interactions. For example:

- **Cancer-focused LD biology reviews published in 2023–2025**
- **Reviews on LD–organelle crosstalk**
- **Reviews discussing LD heterogeneity and lipid metabolism in cancer**

While the current manuscript frames LDs as mediators of cellular homeostasis, the overlap with these publications is substantial. The authors should explicitly state how

their review differs from existing literature and clarify what new conceptual framework it provides.

For example, the manuscript could position itself as a review focused specifically on:

• LDs as stress buffering organelles

• LD-mediated metabolic resilience mechanisms

• LDs linking lipid metabolism and proteotoxic stress

Without this clarification the manuscript risks appearing redundant.

Re: We appreciate this insightful suggestion. Our intent was to write a review on the roles of LDs in buffering stress of cancer cells through interactions with different organelles. To emphasize this topic, we made the following revisions:

1. *We substantially redrew Figures 2 and 3 to highlight LD's dynamic function in buffering ER and mitochondrial redox stress.*
2. *We revised the text of several sections to emphasize that LD dynamics reflect their role buffering stress from nutrient restriction, hypoxia, unfolded proteins, and redox stress.*
3. *To reflect this deeper emphasis on the stress-buffering roles of LDs, we revised the following section titles: 'The role of LDs in buffering nutrient stress in cancer', 'LDs in maintaining oxygen homeostasis under hypoxia in cancer, 'LDs in ER homeostasis and proteostasis', 'LDs in mitochondrial homeostasis under redox stress', 'LDs as buffers of toxin- and cancer therapy-induced stress'.*
4. *In the revised manuscript, we made changes to both the Abstract and the Introduction to more explicitly highlight the focus on LDs as stress-buffering organelles.*
5. *Finally, in light of these revisions, we also changed the overall title of this review to "Lipid Droplets as Stress-Buffering Organelles in Cancer Cell Homeostasis". We believe this revised title clarifies the conceptual and timely emphasis of the review and engages readers.*

Comment 2.2. Insufficient coverage of recent literature

Although the manuscript includes foundational studies, several sections rely heavily on older references and should incorporate more recent work.

In particular, the following areas require updated citations:

• LD-organelle contact sites

- **LD heterogeneity and lipid composition**
- **LDs in immune cells within the tumor microenvironment**
- **LDs in therapy resistance and metabolic plasticity**
- **Methods for studying LD biology**

Recent reviews and studies published in the last 3–5 years should be incorporated to ensure the review reflects the current state of the field.

Re: We thank the reviewer for raising this important point, which helped us improve the timeliness and relevance of this review. In response, we substantially updated the references throughout the manuscript, with particular emphasis on incorporating studies published within the last 5 years. Below are the references we swapped for each topic:

We incorporated the 2024 study 'VHL mutation drives human clear cell renal cell carcinoma progression through PI3K/AKT-dependent cholesteryl ester accumulation' to further support the cancer-specific CE-rich composition of LDs, and to show that this signature is not faithfully retained in conventional cell lines. We also added the 2024 study 'The lipid droplet protein DHRS3 is a regulator of melanoma cell state' to further support that changes in LD-associated proteins are linked to tumor progression, particularly in melanoma.

To strengthen the discussion of ER–LD interactions, we included the 2024 study 'The triglyceride-synthesizing enzyme diacylglycerol acyltransferase 2 modulates the formation of the hepatitis C virus replication organelle', which further suggests that DGAT2-driven LD biogenesis can reshape local ER membrane lipid composition, indicating that LD formation is not only a storage process but may also influence ER membrane lipid flux and phospholipid availability.

For the mitochondrial section, we added the 2024 study 'Perilipin 5 protects the mitochondrial oxidative functions and improves the alcoholic liver injury in mice', which provides more recent support for PLIN5-dependent LD–mitochondria protection against oxidative stress.

For the hypoxia section, we incorporated the 2024 study 'Hypoxia induced cell dormancy of salivary adenoid cystic carcinoma through miR-922/DEC2 axis', which shows in a salivary adenoid cystic carcinoma model, hypoxia-associated miR-922/DEC2 signaling alters LD accumulation, fatty acid oxidation, and DGAT/FASN/CPT1A expression, supporting the idea that hypoxia-driven LD remodeling is context-specific and linked to dormancy and metastasis.

For the section on toxic stress and therapy resistance, we added the 2024 study 'Cancer cells employ lipid droplets to survive toxic stress', which showed in prostate and breast cancer models that LD accumulation increases together with ROS and promotes cell survival through sequestration of damaged or excess lipids. We also included the 2022 study 'AKR1C3-dependent lipid droplet formation confers hepatocellular carcinoma cell adaptability to targeted therapy', which demonstrates that LD formation helps hepatocellular carcinoma cells adapt to the survival stress induced by sorafenib treatment.

At the same time, regarding the include of recent literature on LDs in immune cells within the tumor microenvironment, and in response to your later suggestion to further expand the discussion of LDs in immune responses, we have now separated this topic into an independent paragraph. This new section includes recent studies from the last 5 years on LDs in immune stress adaptation within the tumor microenvironment. The specific details are provided below in our response to the comment on the LD in immune response section.

Together, these additions substantially strengthen the coverage of recent original research and improve the manuscript's representation of the current state of the field.

Comment 2.3. Overgeneralization of mechanistic conclusions

Several sections present findings from specific systems as broadly applicable mechanisms across cancer types.

Examples include:

- **hypoxia-mediated LD accumulation**
- **GPX4 activity and ferroptosis regulation**
- **sequestration of chemotherapeutic agents in LDs**
- **lipid droplet–mediated detoxification**

In many cases these mechanisms have been demonstrated only in particular tumor models or cell lines. The manuscript should clarify when observations are context-specific rather than universal features of cancer biology.

Re: We thank the reviewer for this critique and have revised the manuscript to more explicitly indicate the tumor types, cell models, or experimental systems from which conclusions were drawn. We retained a small number of points supported by non-cancer models that we considered still informative and mechanistically relevant. In addition, in the newly added sections and revised text, we paid particular attention to this issue and more consistently specified the relevant tumor types and cell types wherever appropriate.

Comment 2.4. Mechanistic inaccuracies or imprecise wording

A few statements would benefit from correction or clarification. Examples:

•COX-2 description: The manuscript states that COX-2 catalyzes the synthesis of arachidonic acid into prostaglandins. COX-2 instead converts arachidonic acid into prostaglandin endoperoxides (PGG2/PGH2), which are then converted into prostaglandins by downstream enzymes.

Re: We thank the reviewer for catching this mistake. In the revised review, we clarified that COX-2 converts arachidonic acid into the prostaglandin endoperoxides PGG2 and PGH2, and that these intermediates are then further converted into prostaglandins by downstream enzymes (Nørregaard R, Kwon TH, Frøkiær J. Physiology and pathophysiology of cyclooxygenase-2 and prostaglandin E2 in the kidney. Kidney Res Clin Pract. 2015 Dec;34(4):194–200. doi:10.1016/j.krcp.2015.10.004 PubMed PMID: 26779421). We also state that COX-2 can localize to lipid droplets and support local eicosanoid production.

•FIT2 gene nomenclature

The gene is generally referred to as FITM2, not FIT2M.

Re: Thank you for pointing this out. We have corrected FIT2M to FITM2.

•Lipid droplet growth mechanisms

The manuscript presents coalescence and Ostwald ripening as definitive explanations of LD growth. These processes are plausible biophysical mechanisms but should be described as models rather than universal mechanisms in living cells.

Re: We agree with the reviewer that coalescence and Ostwald ripening should not be presented as definitive or universal mechanisms of LD growth in living cells. We have revised this sentence to describe them more appropriately as proposed biophysical models that may contribute to LD enlargement in certain cellular contexts.

•GPX4 regulation

The statement that oxygen levels directly modulate GPX4 activity is mechanistically vague and should be clarified. Hypoxia influences ferroptosis susceptibility through several pathways, not necessarily direct modulation of GPX4 enzymatic activity.

Re: Thank you for this important comment. We have revised this part to clarify that there is an important relationship between ferroptosis and hypoxia through several pathways, including hypoxia-inducible factor-1 (HIF-1) which is regulated by hypoxia to increase iron uptake, thereby affecting ferroptosis sensitivity. We further clarify that hypoxia influences

ferroptosis susceptibility through changes in iron handling, lipid metabolism, and antioxidant defense, rather than necessarily through direct modulation of GPX4 activity. We also revised the surrounding text to present GPX4 as a core regulator of lipid peroxide detoxification and to specify that LD-mediated buffering of ferroptotic stress has been demonstrated in particular experimental systems rather than as a universal mechanism across all cancers.

Comment 2.5. Missing discussion of experimental methodologies

The manuscript would greatly benefit from a short section describing how lipid droplets are studied experimentally. This addition would increase the utility of the review for readers entering the field.

Possible subsections could include:

Imaging approaches

- **BODIPY / Nile Red staining**
- **confocal microscopy**
- **electron microscopy**
- **correlative light-electron microscopy**

Label-free methods

- **Raman spectroscopy**
- **stimulated Raman scattering imaging**

Biochemical approaches

- **lipid droplet isolation**
- **LD proteomics and lipidomics**
- **isotope tracing for lipid flux**

Functional assays

- **lipolysis assays**
- **lipophagy measurements**
- **organelle contact site analysis**

Such a section would make the review significantly more informative.

Re: We fully agree that a short section summarizing how lipid droplets are studied experimentally would make the review more informative, especially for readers entering the field. In response, we have now added a separate section entitled "Experimental approaches for studying lipid droplets". In revised manuscript, we briefly introduce several major methodological categories commonly used in LD research, including fluorescence imaging, ultrastructural imaging, label-free vibrational imaging, biochemical profiling, metabolic flux analysis, and functional assays. Specifically, the new section now covers:

1.Fluorescence-based imaging approaches, including BODIPY and Nile Red staining combined with widefield or confocal microscopy, as commonly used tools for visualizing LD abundance, size, and intracellular distribution.

2.Ultrastructural methods, including electron microscopy and correlative light-electron microscopy, which are particularly useful for examining LD ultrastructure and physical interactions with organelles such as the ER and mitochondria.

3.Label-free methods, including Raman spectroscopy and stimulated/coherent Raman scattering imaging, which provide chemical information on lipid-rich structures without the need for exogenous dyes.

4.Biochemical approaches, such as LD isolation followed by proteomics or lipidomics, for identifying LD-associated proteins and lipid species.

5.Metabolic flux approaches, such as stable isotope tracing, which can be used to study lipid uptake, synthesis, storage, mobilization, and oxidation.

6.Functional assays, including lipolysis measurements, lipophagy assays, and organelle contact-site analysis, which help connect LD morphology with metabolic activity and stress adaptation.

In this revised text section we did not only list these methods but also emphasized their major strengths and limitations. Moreover, we added "Table 2" summarizing these methods, their representative examples, their main strengths, and their major limitations. We emphasize that no single method is fully sufficient on its own for LD study, as each approach emphasizes different aspects of LD biology. Based on this point, we added a concluding sentence at the end of the section to make this more explicit: "Different methods focus on different aspects of LD biology, including morphology, composition, and function; it is generally best to combine at least two complementary approaches when studying LDs".

We hope this addition makes the review more practical and accessible for readers who are new to the LD study.

Comment 2.6. Tumor microenvironment and immune cells

The role of lipid droplets in immune cells (e.g., tumor-associated macrophages, dendritic cells, T cell exhaustion) is mentioned only briefly in the future directions section. However, LD metabolism in immune cells is now recognized as an important aspect of tumor biology and therapy response. Expanding this discussion would strengthen the translational relevance of the review.

Re: We appreciate this constructive critique. In response, and in line with the reviewer's comment 2.2 regarding the need to update the literature, we added a new paragraph entitled "LDs in immune stress adaptation within the tumor microenvironment". which discusses how LDs may help tumor cells evade immune attack across different cancer types. The key points in this new section are supported by recent original studies published within the last 5 years, including:

A 2024 study 'Protumoral lipid droplet–loaded macrophages are enriched in human glioblastoma and can be therapeutically targeted', which showed that LD-rich macrophage states in glioma are associated with immunosuppressive phenotypes and tumor-promoting functions;

A 2024 study 'Lipid droplets-related Perilipin-3: potential immune checkpoint and oncogene in oral squamous cell carcinoma', which reported that PLIN3-high tumor cells showed increased LD deposition and were associated with reduced CD8+ T-cell activation and greater T-cell exhaustion;

A 2025 study 'Proteogenomic characterization reveals that lipid droplet formation promotes esophageal squamous cell cancer progression', which showed that in esophageal squamous cell carcinoma, PLIN2/APOE-driven LD formation promoted cytokine secretion, stromalization, and suppression of T-cell activation.

To better present and summarize this newly added content, we also included a new figure (Figure 4) to visualize these findings.

Minor Comments

1. Several sections could benefit from clearer transitions to improve narrative flow.

Re: We have revised the language in several sections to improve transitions between paragraphs and strengthen the overall narrative flow of the manuscript.

2. The manuscript should distinguish between neutral lipid storage (TAG vs cholesteryl esters) and their distinct roles in cancer metabolism.

Re: We have revised the section "LDs biosynthesis in cancer" to more clearly distinguish between neutral lipid storage in the form of TAGs versus cholesteryl esters, and to better explain their distinct roles in cancer metabolism.

3. The future directions section is somewhat generic. It could be improved by highlighting specific unresolved questions, such as:

o LD heterogeneity across cancer types

o lipid droplet–mediated drug resistance mechanisms

o LD involvement in ferroptosis and oxidative lipid metabolism

Re: We revised the Future Directions section to make it more focused on concrete unresolved questions in the field. In the revised version, we now explicitly highlight several major open areas, as requested by the reviewer, explaining why each represents an important biological and translational gap. In addition, we provide a specific example of a therapeutic approach targeting the DGAT pathway in non-oncology settings.

4. Figures illustrating LD-organelle interactions or metabolic pathways would improve clarity.

Re: Thank you for this helpful suggestion. We have revised the Graphic Abstract to more clearly and comprehensively illustrate LD–organelle interactions. In addition, each of the main figures in the manuscript (Figures 1–3) has been further updated, with the relevant pathways and mechanistic relationships now presented in greater detail to improve clarity and better match the corresponding text.

Suggested Additional Topics

The manuscript would benefit from including discussion of:

• lipid droplet heterogeneity

• spatial lipidomics and LD composition

• LD–peroxisome interactions

• LD regulation of ferroptosis

• lipid droplets in metastatic cancer cells

• metabolic plasticity during therapy resistance

Re: Following the reviewer's suggestion, we tried to incorporate as many of these additional points as possible, focusing on recently published work and citing important review papers

on these topics, whenever available, to avoid redundance. Given our expertise in central nervous system cancers, we specifically emphasize the work in cancers.

Lipid droplet heterogeneity: An excellent review on this topic was published earlier this year (Henne, W.M., Cohen, S. Heterogeneity, dynamics and organelle interactions of lipid droplets. Nat Rev Mol Cell Biol (2026). https://doi.org/10.1038/s41580-025-00945-x), inspired by this review, we strengthened the discussion of LD heterogeneity and compositional diversity in the section on structural and functional remodeling of LDs and future dirctions.

Spatial lipidomics and LD composition: We added recent evidence indicating LD composition heterogeneity across tumor regions and cellular states, thereby introducing the spatial aspect of LD composition. Specifically, we review work from the following manuscripts:

- *Hariri H, Speer N, Bowerman J, Rogers S, Fu G, Reetz E, et al. Mdm1 maintains endoplasmic reticulum homeostasis by spatially regulating lipid droplet biogenesis. J Cell Biol. 2019 Feb 26;218(4):1319–34. doi:10.1083/jcb.201808119*
- *Zhang S, Fang T, He Y, Feng W, Yu Z, Zheng Y, et al. VHL mutation drives human clear cell renal cell carcinoma progression through PI3K/AKT-dependent cholesteryl ester accumulation. EBioMedicine. 2024 May;103:105070. doi:10.1016/j.ebiom.2024.105070 PubMed PMID: 38564827; PubMed Central PMCID: PMC10999658.*

For LD–peroxisome interactions, the currently available evidence is relatively limited, and the most direct mechanistic study is not based in a cancer context. Therefore, we did not expand this topic into a separate subsection, but we mentioned it in the Discussion as a potentially relevant but still underexplored component of LD–organelle crosstalk.

LD regulation of ferroptosis. We thank the reviewer for this suggestion. In 2023, Dr. Pope and Dixon wrote an excellent review entitled "Regulation of ferroptosis by lipid metabolism" (Trends in Cell Biology, 2023), We therefore only discuss crucial manuscripts on this topic published between 2023-2026. Specifically, we review work from the following manuscripts:

- *Lee H, Horbath A, Kondiparthi L, Meena JK, Lei G, Dasgupta S, et al. Cell cycle arrest induces lipid droplet formation and confers ferroptosis resistance. Nat Commun. 2024 Jan 2;15(1):79. doi:10.1038/s41467-023-44412-7 PubMed PMID: 38167301; PubMed Central PMCID: PMC10761718.*

- *Cao R, Feng Z, Mo J, Wu J, Li J, Li W, et al. Pharmacological inhibition of SREBP1 suppresses pancreatic cancer growth via inducing GPX4-mediated ferroptosis. Cellular Signalling. 2024 Dec 1;124:111381. doi:10.1016/j.cellsig.2024.111381*
- *Han L, Wang Z, Jiang Y, Zhou H, Zhou H. Ferroptosis and metabolic reprogramming in the immunosuppressive microenvironment of glioblastoma: emerging mechanisms and novel strategies. Front Immunol. 2026 Apr 14;17:1775148. doi: 10.3389/fimmu.2026.1775148.*

In addition, to address the relevance of LDs in metastatic cancer cells and therapy adaptation, we expanded the discussion of LD remodeling in aggressive tumor states by adding example research linking LD formation to metastatic behavior. Specifically, we review work from the following manuscripts:

- *Liu Y, Shen L, Li Y, Sun X, Liang L, Jiang S, et al. ETS1-mediated Regulation of SOAT1 Enhances the Malignant Phenotype of Oral Squamous Cell Carcinoma and Induces Tumor-associated Macrophages M2-like Polarization. Int J Biol Sci. 2024 Jun 11;20(9):3372–92. doi:10.7150/ijbs.93815 PubMed PMID: 38993570; PubMed Central PMCID: PMC11234219.*
- *Corbet C, Bastien E, Santiago de Jesus JP, Dierge E, Martherus R, Vander Linden C, et al. TGFβ2-induced formation of lipid droplets supports acidosis-driven EMT and the metastatic spreading of cancer cells. Nat Commun. 2020 Jan 23;11(1):454. doi:10.1038/s41467-019-14262-3*

And by adding a discussion section under toxic management, we explicitly stating that LD remodeling can support metabolic plasticity during therapy resistance through drug sequestration, buffering of toxic lipids, and survival adaptation under treatment stress. Specifically, we review work from the following manuscripts:

- *Verbrugge SE, Al M, Assaraf YG, Kammerer S, Chandrupatla DMSH, Honeywell R, et al. Multifactorial resistance to aminopeptidase inhibitor prodrug CHR2863 in myeloid leukemia cells: down-regulation of carboxylesterase 1, drug sequestration in lipid droplets and pro-survival activation ERK/Akt/mTOR. Oncotarget. 2015 Oct 19;7(5):5240–57. doi:10.18632/oncotarget.6169 PubMed PMID: 26496029; PubMed Central PMCID: PMC4868683.*
- *Wu C, Dai C, Li X, Sun M, Chu H, Xuan Q, et al. AKR1C3-dependent lipid droplet formation confers hepatocellular carcinoma cell adaptability to targeted therapy. Theranostics. 2022;12(18):7681–98. doi:10.7150/thno.74974 PubMed PMID: 36451864; PubMed Central PMCID: PMC9706585.*

Reviewer #3 (Remarks to the Author):

Major:

Comment 3.1. Explanation of the key biochemical pathways leading to neutral lipid synthesis would be very beneficial for the paper, as many of the enzymes are mentioned in the review but their role in lipid and LD metabolism is not explained.

Re: We addressed this point by revising the section on LD biogenesis in cancer to more clearly explain the key biochemical pathways leading to neutral lipid synthesis. In the revised manuscript, we now describe how ACLY, ACC, and FASN support fatty acid synthesis, how AGPAT2 and Lipin-1 contribute to glycerolipid intermediate formation, how DGAT1/2 catalyze the final step of TAG synthesis, and how SOAT1 generates cholesteryl esters from free cholesterol. We believe this addition makes the roles of these enzymes in lipid and LD metabolism much clearer under this version.

Comment 3.2. Figure 1: The lines/pathways connecting the various enzymes and elements are not clear.

"How are DGAT1/2 and ACLY directly linked? Why does FA uptake through CD36 proceed via the nucleus?"

Re: Figure 1 has been substantially redesigned. The connections between the different regulators and metabolic steps are now shown more clearly. In the revised figure, fatty acid uptake no longer passes through the nucleus. The meaning of "lipids and fatty acids," as well as the related synthetic steps, are now more specifically illustrated.

"Lipids and FAs" are produced by FASN? Are the lipids depicted as small droplets somehow involved in generating bigger droplets?"

Re: Small lipid droplets usually contribute to the formation of the main lipid droplet through Ostwald ripening, as discussed in the text, while the main lipid droplet can also undergo degradation into smaller lipid droplets, these regulatory processes are indicated in the revised figure now. We also cite an excellent review that describes the life cycle of a lipid droplet in great detail: 'Henne, W.M., Cohen, S. Heterogeneity, dynamics and organelle interactions of lipid droplets. Nat Rev Mol Cell Biol (2026). https://doi.org/10.1038/s41580-025-00945-x'.

Comment 3.3. The chapter on the "molecular composition of LDs" should be revised/expanded to clarify and specify in more detail how is the lipid/protein composition of LDs altered in cancer. For example, it is not clear from the text how and which changes in DGAT1/2 are cancer-specific? The relevance of the findings that eicosanoids are present at the surface of LDs should be clarified. Does eicosanoid

production occur at the LD surface or at the ER membrane nearby? Which LD enzymes are involved? Are these oxylipins released from cells? Is this mechanism cancer-specific?

Re: We revised this chapter to clarify in more concrete terms how LD composition is altered in cancer, First, we expanded the text to better distinguish lipid and protein remodeling of LDs in cancer, including that LDs can differ in neutral lipid composition, associated protein cargo, and spatial distribution across tumor regions and cellular states (Revised manuscript, page x, line x).

"For example, it is not clear from the text how and which changes in DGAT1/2 are cancer-specific?"

Re: with focus on reviewer's points on DGAT1/2 and LD-associated eicosanoid production. We also clarified that changes in DGAT1/2 should be viewed as context-dependent rather than universal across all cancers. In the revised version, we now explain more explicitly that DGAT1/2 are not simply generic LD markers, but enzymes that can be upregulated or functionally engaged in tumor settings with high lipid flux, where they support TAG synthesis, LD expansion, and stress adaptation.

"The relevance of the findings that eicosanoids are present at the surface of LDs should be clarified. Does eicosanoid production occur at the LD surface or at the ER membrane nearby? Which LD enzymes are involved? "

Re: Second, we revised the eicosanoid-related paragraph to make the mechanism and its relevance clearer. We now specify that COX-2 converts arachidonic acid into PGG2 and PGH2, and we cite original studies showing that, in colon cancer cells, lipid bodies can act as reservoirs of COX-2 and as sites of PGE2 synthesis. We also note that coordinated induction of LD biogenesis and the COX-2 pathway has been reported in Barrett's esophagus and esophageal adenocarcinoma models, and that more recent work supports a role for LD biogenesis in mitogenic lipid mediator production in human cancer cells (revised manuscript, page x, line x). Based on these revisions, the text now clarifies that at least part of eicosanoid production can occur at LD-associated structures in specific models, rather than being assumed to occur universally or exclusively at nearby ER membranes.

"Are these oxylipins released from cells?"

Re: Although prostaglandins such as PGE2 have clear autocrine/paracrine signaling relevance, we did not identify sufficiently direct evidence showing release of LD-associated oxylipins from cancer cells in the specific contexts discussed here. Therefore, we did not add a stronger statement or citation on this point in the revised manuscript.

"Is this mechanism cancer-specific?"

We also adjusted the wording to avoid overgeneralization. In particular, we now make clear that LD-associated eicosanoid production should be regarded as context-dependent rather than a universal feature across all cancers. In addition, rather than implying that all downstream enzymes are established as LD-resident in every setting, we now frame this more carefully around the demonstrated COX-2-associated prostaglandin pathway and its functional consequences. Because prostaglandins such as PGE2 are established signaling mediators, we now clarify their relevance in the context of tumor-promoting lipid signaling rather than leaving their functional significance implicit. We believe these changes make this section more mechanistically precise and more responsive to the reviewer's concerns.

Comment 3.4. Line 107: Could the authors provide a reference that links mTOR with lipophagy and not lipolysis?

Re: We revised the wording of the mTOR-related sentence to make it more accurate. In addition, to address this, we have now added a more direct reference linking mTOR signaling to lipophagy (Garcia-Macia M, Santos-Ledo A, Leslie J, Paish HL, Collins AL, Scott RS, et al. A Mammalian Target of Rapamycin-Perilipin 3 (mTORC1-Plin3) Pathway is essential to Activate Lipophagy and Protects Against Hepatosteatosis. Hepatology. 2021 Dec;74(6):3441–59. doi:10.1002/hep.32048 PubMed PMID: 34233024.). This study shows that mTORC1 regulates lipophagy through Plin3-dependent mechanisms.

Comment 3.5. The authors should clarify how lipin-1 and AGPAT2 "catalyze TAG synthesis"?

Re: We revised this sentence to make the roles of Lipin-1 and AGPAT2 more biochemically precise. In the revised text, we no longer state that they directly catalyze TAG synthesis as a single step. Instead, we now clarify that AGPAT2 and Lipin-1 participate in the TAG biosynthetic pathway at intermediate steps, with AGPAT2 promoting phosphatidic acid formation and Lipin-1 converting phosphatidic acid to diacylglycerol, the direct precursor of TAG (Triantafyllou EA, Georgatsou E, Mylonis I, Simos G, Paraskeva E. Expression of AGPAT2, an enzyme involved in the glycerophospholipid/triacylglycerol biosynthesis pathway, is directly regulated by HIF-1 and promotes survival and etoposide resistance of cancer cells under hypoxia. Biochim Biophys Acta Mol Cell Biol Lipids. 2018 Sep;1863(9):1142–52. doi:10.1016/j.bbalip.2018.06.015 PubMed PMID: 29908837.). We also retained the hypoxia-specific references showing that both enzymes are regulated in this context.

Comment 3.6. Lines 144-146: This section should be clarified. Could the authors provide more details on how cancer cells divert PUFAs into LDs? The referenced study

by Magtanong et al only addressed the role of exogenous MUFAs in ferroptosis, but there is indeed ample evidence that PUFA storage is protective against ferroptosis, but also other forms of lipotoxicity.

Re: We revised this section to clarify the mechanism more precisely. We now explain more explicitly that increased DGAT1/2 activity promotes TAG synthesis and LD biogenesis, thereby allowing excess and oxidizable fatty acids to be incorporated into neutral lipid stores. We further clarify that this process is thought to reduce the availability of peroxidation-prone fatty acids in membrane phospholipids and can therefore buffer ferroptotic stress as well as broader lipotoxic stress.

Comment 3.7. Table 1 is interesting but should provide key references for each statement.

Re: Thank you for this helpful suggestion. We have redesigned Table 1 by adding one column describing the consequences for cancer cells and another column listing references for each statement. At the same time, we also revised the "Adaptations on LDs" column to make the wording more precise and rigorous.

Comment 3.8. Fig. 2: The figure should be thoroughly revised as the graphical elements are misleading and not clear. Why is a TAG molecule depicted near Rab18? Which MCS sites are depicted? Does palmitic acid block ER stress as suggested? Is there a shuttle from the ER to LDs?

Re: Thank you for these helpful comments. We agree that the previous Figure 2 was not clear enough in several parts. We have therefore completely redrawn Figure 2. In the revised figure, the ER–LD interaction is shown more clearly and in a more logical way, including the membrane contact sites (MCSs) and the proteins involved in these contact sites. We also now show the physical connection between the ER and LDs more explicitly, and clarify the dynamic movement of DGAT2 and acyl-CoA synthetases along the ER–LD interface through MCS. In addition, we corrected the previous presentation of palmitic acid and UPR and related ER stress pathway. The revised figure now better illustrates that potentially lipotoxic fatty acids such as palmitate are esterified and stored in LDs, thereby reducing their accumulation in ER membranes and helping to limit ER stress, rather than directly suggesting that palmitic acid itself blocks ER stress.

Comment 3.9. Fig legends should contain short descriptions of the depicted pathways and mechanisms.

Re: We revised all figure legends throughout the manuscript to include short descriptions of the main pathways and mechanisms depicted in each figure. In particular, the updated legends now briefly explain the biological processes illustrated in Figures 1–4 and also

graphic abstract, including LD biogenesis, ER–LD interactions, LD–mitochondria buffering, and LD-mediated immune stress adaptation. We believe these changes improve clarity and make the figures easier to interpret together with the main text.

Comment 3.10. Fig. 3. TG molecule is labeled as FA. Which is the complex lipid presented on top, containing 5 FA chains and two headgrops? Are TGs molecules transported through channels on the plasma membrane?

Re: Thank you for these helpful comments. We have thoroughly revised this figure. In the new version, free fatty acids, TAG storage within LDs, and controlled fatty acid transfer to mitochondria are more clearly distinguished. We removed the misleading presentation suggesting that TG is transported through plasma membrane channels, and corrected the lipid structures shown in the schematic. We also added more mechanistic detail to illustrate that excess fatty acids can be re-esterified and stored in LDs, followed by controlled transfer to mitochondria through LD–mitochondria contact, thereby limiting ROS accumulation and preserving mitochondrial function.

Comment 3.11. Lines 212-216: Revise to clarify how is LD PUFA storage mechanistically linked to nuclear homeostasis?

Re: We revised this section to clarify that the connection between LD-mediated lipid sequestration and nuclear homeostasis is indirect rather than a direct nuclear mechanism. In the revised text, we now explain that therapy-induced cell-cycle arrest is a nucleus-centered process, and that subsequent cell-cycle progression requires preservation of overall cellular and nuclear stability.

Comment 3.12. Line 222: Are LDs biomolecular condensates?

Re: We agree that LDs should not be described as biomolecular condensates. Our intention was only to compare their physical properties, not to classify them as condensates. To avoid this ambiguity, we revised the sentence and removed the comparison to biomolecular condensates. In the revised text, we now state more directly that LDs have sufficient mechanical rigidity to deform surrounding cellular structures and thereby stress the nucleus.

Comment 3.13. Line 261: The prospect of targeting LDs for therapy should be discussed in the context of numerous existing preclinical studies using DGAT inhibitors in mice or humans, or other LD targets, and not FASN inhibitors, which inhibit de novo FA synthesis and do not block LD metabolism per se.

Re: We have revised the Future Directions section to discuss LD-targeted therapy more specifically in the context of DGAT-dependent LD biogenesis and other LD-related

pathways. In the revised manuscript, we now clarify that the DGAT pathway is more directly linked to neutral lipid synthesis and LD formation, and we note that DGAT-directed strategies have already been explored therapeutically in non-oncology settings in Phase II clinical trial, may supporting the druggability of this axis under cancer in the future. We also revised the paragraph to better distinguish inhibition of LD formation, turnover, or stress-buffering function from broader inhibition of upstream de novo fatty acid synthesis, and to emphasize that future translation into cancer will require attention to tumor specificity, tolerability, and rational combination design.

Minor: Abbreviations are not defined. And when defined they are not consistently used. And some abbreviations are defined multiple times. All abbr. in figures should be defined.

Re: We carefully revised the abbreviations throughout the manuscript to improve consistency and clarity. In the revised version, abbreviations are now defined more systematically at first appearance, repeated or inconsistent definitions have been corrected, and the abbreviation list has been reorganized and updated accordingly.

Fig. 2: what is Acyl-Coa syn?

Re: Thank you for pointing this out. We agree that this abbreviation was not presented in a standard form. In the revised figure, we have corrected "Acyl-CoA syn" to the full term "acyl-CoA synthetases."

COMMSBIO_26-1213B

Dear Editor Georgieva and Reviewers,

We sincerely thank you for your careful evaluation of our manuscript and for the constructive and insightful comments. These suggestions have been highly valuable in improving the clarity, rigor, and overall quality of our review. Below, we provide a point-by-point response to all comments. Reviewer comments are shown in **bold**, and our responses are shown in *italics*.

Reviewers' comments:

Reviewer #3 (Remarks to the Author):

Comment 1.1. In several figures ACSS2 is depicted as acting on fatty acids (and activating them??) taken up through CD36. As far as I know, this enzyme is very specific for acetate, driving de novo lipogenesis... The whole downstream pathway leading to LD accumulation in these figures therefore does not make any sense.

Re: We thank the reviewer for pointing out these important inaccuracies. We agree that the previous version of Figure 1 contained oversimplified and, in several places, misleading depictions of lipid metabolic pathways and lipid structures. Our intention was to include multiple cancer-associated routes leading to lipid droplet accumulation in a compact schematic; however, this compression resulted in inaccurate pathway representation and could cause misunderstanding. We have therefore substantially revised and expanded Figure 1.

We removed the misleading depiction of ACSS2 as an enzyme acting downstream of CD36-mediated fatty acid uptake. In the revised figure, ACSS2 is shown specifically in the acetate-utilization pathway, converting acetate to acetyl-CoA, which can then contribute to de novo lipogenesis. In contrast, exogenous fatty acids taken up through CD36 are now shown to be activated by long-chain acyl-CoA synthetases, including the ACSL family, before entering downstream neutral lipid synthesis.

Comment 1.2. TGs are depicted with a benzene ring and 5 acyl chains... Cholesterol also contains benzene and 2 chains apparently? In figure 1, these TGs, CEs are depicted in the cytosol and away from lipid droplets, which are shown a bit lower. Why?

Re: We revised the lipid droplet biogenesis pathway to more accurately show neutral lipid synthesis at the endoplasmic reticulum. The revised schematic now depicts fatty

acyl-CoA as a substrate for DGAT1/2-mediated triacylglycerol synthesis and SOAT1-mediated cholesterol esterification, producing triacylglycerols and cholesteryl esters that are stored in the lipid droplet core.

We then corrected the lipid structures and their localization. The previous cartoon symbols for triacylglycerols, cholesteryl esters, and cholesterol were inaccurate and could be interpreted as benzene-containing molecules with incorrect numbers of acyl chains. These structures have been removed/replaced with simplified but chemically appropriate schematic representations. We believe these revisions address the reviewer's concern and make the figure more biologically and chemically accurate.
